# Rapid centromere turnover and the adaptive radiation of lemurs

**DOI:** 10.64898/2026.05.16.725662

**Published:** 2026-05-19

**Authors:** Mihir Trivedi, Francesca Gianfrate, Luciana de Gennaro, Marcelo Ayllon, Katherine M. Munson, Kendra Hoekzema, DongAhn Yoo, Erin E. Ehmke, Anne D. Yoder, Stephen Chang, Chinmay Lalgudi, Mark Krasnow, Mario Ventura, Evan E. Eichler

**Affiliations:** 1 Department of Genome Sciences, University of Washington School of Medicine, Seattle, WA, USA.; 2 Howard Hughes Medical Institute, University of Washington, Seattle, WA, USA.; 3 Department of Biosciences, Biotechnology and Environment, University of Bari Aldo Moro, Bari, Italy.; 4 Duke Lemur Center, Durham, NC, USA.; 5 Department of Biology, Duke University, Durham, NC, USA.; 6 Department of Biochemistry, Stanford University School of Medicine, Stanford, CA, USA.; 7 Howard Hughes Medical Institute, Stanford, CA, USA.

## Abstract

Centromeres represent essential chromosomal structures required for faithful chromosome segregation during cell division but are paradoxically hypermutable, leading to centromere drive and reproductive isolation in closely related species. Using long-read sequencing, we generate nearly complete genomes (2.1–2.5 Gbp) from eight lemur species and characterize the sequence, epigenetic and cytogenetic structure of 223 strepsirrhini centromeres providing an alternative primate perspective of centromere evolution. No lemur centromere consists of α-satellite DNA that typifies the haplorhine lineage; instead, each species evolved its own distinct higher-order centromeric repeat sequence, varying substantially in both monomer length (ranging from 41–548 bp) and primary sequence composition (GC percentages 28.7–67.9%) including centromere cooption of telomeric repeats in brown lemurs. Most centromeres show characteristic hypomethylation dip regions (110–300 kbp) as candidates for kinetochore attachment. The centromere sequence motif shows no apparent sequence homology among lemur genera, even for species separated by less than 15 million years (*Lemur* and *Eulemur*). We estimate a >6-fold increased rate in primary centromeric motif turnover in strepsirrhines when compared to haplorhines and this occurred in conjunction with positive selection of the CENP-B protein in lemur lineages. We propose that lemur radiation and centromere diversification are linked, whereby accelerated motif turnover provides a stasipatric barrier contributing to rapid chromosomal evolution.

## INTRODUCTION

At the most basal level, the primate order is classified into two clades: strepsirrhines and haplorhines. The suborder Strepsirrhini is further comprised of lemurs (Lemuriformes), lorises (Lorisidae), and galagos (Galagidae) (Fleagle, 2013). Lemuriformes have been a focus of primate research due to their diversity and a restricted geographical distribution, which is limited to the island of Madagascar (Yoder, 2007). With some estimates of species diversity ranging as high as 112 extant and 17 extinct species concentrated in less than 15% of the planet’s land area, lemurs are uniquely well suited to provide insights into animal speciation (Mittermeier et al., 2022; Godfrey et al., 2010). They are thought to have diverged from the clade containing Old World and New World haplorhine primates 70–79 million years ago and represent the deepest split among the primates, rendering them critical to understand early primate adaptations (Horvath & Willard, 2007; dos Reis et al., 2018).

Owing to this complicated diversification, the evolutionary history of lemurs and the forces that shaped their speciation remain a matter of debate. A wealth of karyotypic data has been gathered over the last 50 years for all major lineages within the Lemuriformes, including the Lemuridae, Indriidae, Cheirogaleidae, and monotypic aye-aye, *Daubentonia* (Rumpler & Dutrillaux, 1976; Rumpler & Dutrillaux, 1979; Rumpler et al., 1983; Rumpler et al., 1988). Ultimately, this work culminated with informed speculation on the ancestral karyotype for the lemuriform clade as well as the sequence of chromosomal fissions and fusions that produced the lineage-specific karyotypes of living lemurs (Warter et al., 2005). While lemur phylogeny and evolution are still an active area of research (Everson et al., 2025a; Orkin et al., 2025), it is largely agreed that Madagascar’s environmental heterogeneity must have provided a variety of niches for ancestral lemur species to speciate after their oceanic dispersal from mainland Africa (Samonds et al., 2012; Yoder et al., 1996).

Despite their central role in understanding primate evolution, there is a paucity of high-quality genomes, and regions such as centromeres have largely been uncharacterized. Among the publicly available reference quality lemur genomes from 41 species, 38 are derived from Illumina short reads (Guevara et al., 2021; Kuderna et al., 2023), while three recent genomes incorporate PacBio and/or Oxford Nanopore Technologies (ONT) long reads (Everson et al., 2025b; Palmada-Flores et al., 2022; Versoza & Pfeifer, 2024). Most of these are highly fragmented, with NCBI labeling seven assemblies as “chromosome level.” Some lemur genomes remain unpublished with preliminary analysis done either independently or as a part of sequencing consortia (Supplementary Table 1). Many of these early assemblies are limited by their low contiguity and high numbers of unplaced scaffolds, rendering them insufficient for the study of centromere structure and evolution.

Recent advances in deep long-read sequencing from both ultra-long ONT as well as PacBio high-fidelity (HiFi) coupled to algorithmic improvements (Cheng et al., 2021), such as the Verkko assembler (Antipov et al., 2025), have resulted in much longer contiguous assemblies traversing some of the longest repeat repetitive regions of primate genomes (Nurk et al., 2022), including centromeres (Logsdon et al., 2024; Logsdon et al., 2025). In order to systematically characterize centromeres among the Strepsirrhini, we focused on the generation of *ab initio* long-read assemblies of eight species. We use these new assemblies to focus on the genetic characterization of lemur centromeres. To date, only the gray mouse lemur and the aye-aye centromeres have been characterized (Larsen et al., 2017; Lee et al., 2011). The former centromere consists of a 53 bp motif (Mm53) that is tandemly repeated to create complex arrays from 400 kbp to 3.2 Mbp, depending on the chromosome. The aye-aye was reported to have two consensus sequences of 146 bp and 268 bp. We extend this work to a newer assembly of the gray mouse lemur and seven other lemuriforms representing 40 million years of diversity. Here, we describe differences in the primary repeat motifs and relative chromosomal location in the context of species-specific higher-order repeat (HOR) structure as well as epigenetic properties associated with the hypomethylation centromere dip region (CDR) thought to define the location of the kinetochore binding among humans and other primates (Logsdon et al., 2024; Yoo et al., 2025).

## RESULTS

### Lemur genome assembly and synteny breakpoints.

**Figure 1 |:**
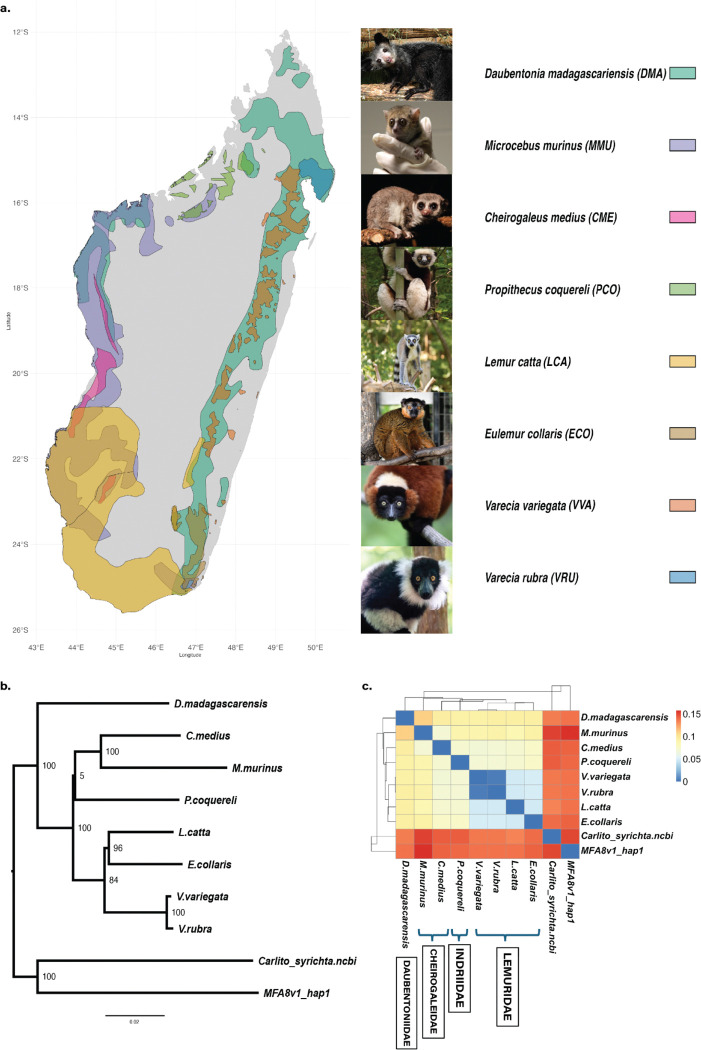
Lemur species and their phylogenetic relationships. a, The geographic distribution of lemur species, used in this study, on the island of Madagascar per the latest release of the International Union of Conservation of Nature (IUCN). The color scheme is described with scientific names and their three letter codes used in the paper. b, Phylogenetic relationship among species calculated based on genetic distances (Methods), using *Macaca fascicularis* and *Carlito syrichta* sequences as the outgroups. Bootstrap values are shown for each node. c, Heatmap showing the pairwise distances between species and delineating four primate families represented by the eight species.

We selected eight lemur species representing both short and long genetic distances over the last 40 million years of strepsirrhine evolution (Everson et al., 2025a): *Lemur catta* (LCA, ring-tailed lemur), *Microcebus murinus* (MMU, gray mouse lemur), *Cheirogaleus medius* (CME, fat-tailed dwarf lemur), *Propithecus coquereli* (PCO, Coquerel’s sifaka), *Eulemur collaris* (ECO, collared brown lemur), *Varecia rubra* (VRU, red ruffed lemur), *Varecia variegata* (VVA, black and white ruffed lemur), and *Daubentonia madagascariensis* (DMA, aye-aye) (Figure 1a). With the exception of *M. murinus*, which was assembled independently (Methods), we obtained primary blood lymphocytes for the other seven species from the Duke Lemur Center (DLC) and extracted high molecular weight DNA. The same material was sequenced with both long-read technologies, PacBio HiFi and ONT, to obtain high coverage for each species (median coverages of 64x and 34x, respectively [Table 1]). For the purpose of centromere reconstruction (often associated with Mbp of tandem repeats), we focused on the production of ultra-long ONT reads to effectively span such regions of the genomes.

In general, the assemblies were generated without chromosomal-level phasing since our focus was to locally assemble centromeres, with two exceptions. For two species, *Lemur catta* (LCA) and *Propithecus coquereli* (PCO), we collected parental samples and phased the assembly with parental Illumina short-read whole-genome sequences. All non-parental phased assemblies were generated by hifiasm (version 0.19.9 or 0.23.0) (Cheng et al., 2021), while parentally phased assemblies were generated with both hifiasm and Verkko (2.1.2) (Antipov et al., 2025), and the best out of the two was chosen based on contiguity and other parameters. By using these techniques, we successfully generated highly contiguous assemblies, each featuring multiple near-telomere-to-telomere (T2T) contigs across all species studied (Supplementary Figure S1).

To confirm the genetic relationship among species, we computed the genetic distances among the genomes to construct a bootstrapped neighbor-joining tree using Mashtree (Katz et al., 2019; Ondov et al., 2016). For phylogenetic reconstruction, we selected the crab-eating macaque, *Macaca fascicularis* (MFA) T2T genome (Zhang et al., 2025), and the tarsier, *Carlito syrichta* (CSY) (Schmitz et al., 2016) genome, as outgroups. The phylogenetic tree supports the generally accepted phylogeny of the taxa, including the assignment of congeneric species (Everson et al., 2025a); however, the split between *Propithecus* and Cheirogaleidae (*Microcebus* and *Cheirogaleus*) was not confidently resolved as evidenced by the lower bootstrap support (Figure 1b). The unresolved nature of the *Propithecus* genus and the overall Indriidae family among other lemur families has been previously described (Harrera and Davalos, 2016). Aye-aye (*Daubentonia*) is the clear outgroup of all the lemur species, while true lemurs (Lemuridae) form the innermost cluster.

**Figure 2 |:**
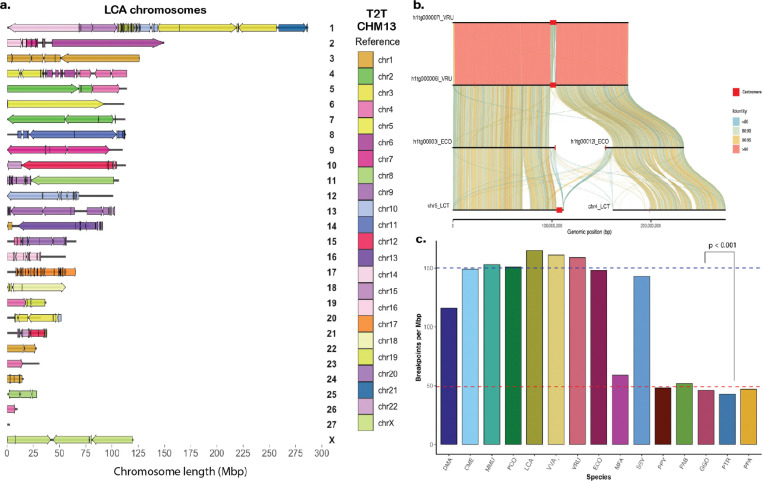
Chromosome level assembly and synteny breakpoints. a, Synteny between T2T-CHM13 and *Lemur catta* (LCA) chromosomes, with LCA on the left and human (T2T-CHM13) chromosome colors on the right. Arrows show the orientation of T2T-CHM13 synteny blocks and straight lines show the regions with no syntenic matches. b, Individual contig/chromosome level synteny with SVbyEye. Single contig from two sister species, VRU and VVA, aligns to two different contigs in ECO and two different chromosomes in LCA with breakpoints occurring over a centromere (red). The color scheme shows % identity between aligned sequences with >95% between VRU and VVA. c, Comparison of the total number of syntenic breakpoints of the lemur species in this study and nonhuman apes (Yoo et al., 2025) compared to human (T2T-CHM13). The mean number of non-human apes’ breakpoints (red) and lemur syntenic breakpoints (blue) is compared p < 0.001.

We also aligned the eight assembled strepsirrhine genomes to the finished human reference genome, T2T-CHM13, using Anchorwave aligner (Song et al., 2022) (Methods). Given their deep divergence, strepsirrhines are expected to have a large number of chromosomal rearrangements with respect to other haplorhine primates. With respect to humans, *Lemur catta* (LCA) has 159 synteny breakpoints. Five chromosomes from T2T-CHM13: 1, 7, 12, 22 and X, are fully conserved with respect to synteny, albeit with intrachromosomal rearrangements and changes in gene order (Figure 2a). As a control, we also performed a similar synteny analysis comparing LCA against the recently completed genome of MFA (Zhang et al., 2025) (Supplementary Figure S2).

We consolidated breakpoint counts across three primate groups: eight strepsirrhines, eight Old World monkeys and apes, and the human T2T-CHM13 reference. A representative syntenic segment across four closely related species, LCA, ECO, VVA, and VRU, illustrates the disruption of sequence identity and collinearity among lineages that diverged approximately 15 million years ago (Figure 2b). As expected, we observe a significant difference (p<0.0001, Wilcoxon-Rank test) between number of breakpoints when comparing strepsirrhines and haplorhines to human or macaque (Figure 2c, Supplementary Figure S3). Overall, there is a gradual decline in the number of syntenic blocks as a function of genetic distance with the conspicuous exception of siamang (SSY), which is known to have undergone an exceptionally rapid karyotype evolution among the apes (Carbone et al., 2014, Yoo et al., 2025). The aye-aye (DMA) shows the lowest number of breakpoints when compared to complete haplorhine genomes consistent with its rather conserved karyotype of 2n = 30 (Poorman-Allen and Izzard, 1990).

### Lemur centromere motif discovery, validation and characterization.

Since α-satellite motifs that are organized into HOR satellite arrays typically define centromeres in haplorhine primates (Yoo et al., 2025), we specifically searched within contiguous genome assemblies for the presence of long tandem repeats using Tandem Repeats Finder (TRF) (Benson, 1999). For each genome, we identified several potential candidates. Next, we designed a set of in-house scripts to then characterize a consensus repeat and used these to define potential higher-order structures on each chromosome as well as their distribution among chromosomes within each species. This is similar to methods used previously to define centromeres in mouse lemur and other mammal species (Melters et al., 2013; Larsen et al., 2017).

Employing this approach, we identified 223 potential centromeres from a total of ~432 lemur contigs. Of note, not all chromosomes were sequenced completely and the karyotype for ECO is not precisely known. Nevertheless, we identified 10 distinct monomer motifs for seven strepsirrhine species (excluding Mm53), including both high GC and high AT-repeat motifs (Figure 6 and Table 2). We designated each using the convention previously applied to *Microcebus murinus*; namely, species acronym followed by motif length).

Overall, our analysis shows that the basic strepsirrhine centromere motifs differ by 10-fold in length ranging from 41–548 bp (Table 2). Perhaps surprisingly, all species belonging to different genera harbor their own specific centromeric monomer sequence motifs. This suggests higher rates of centromere turnover than that observed for haplorhines. Both congeneric species belonging to *Varecia*, in contrast, share the same monomer repeat unit (Table 2).

**Figure 3 |:**
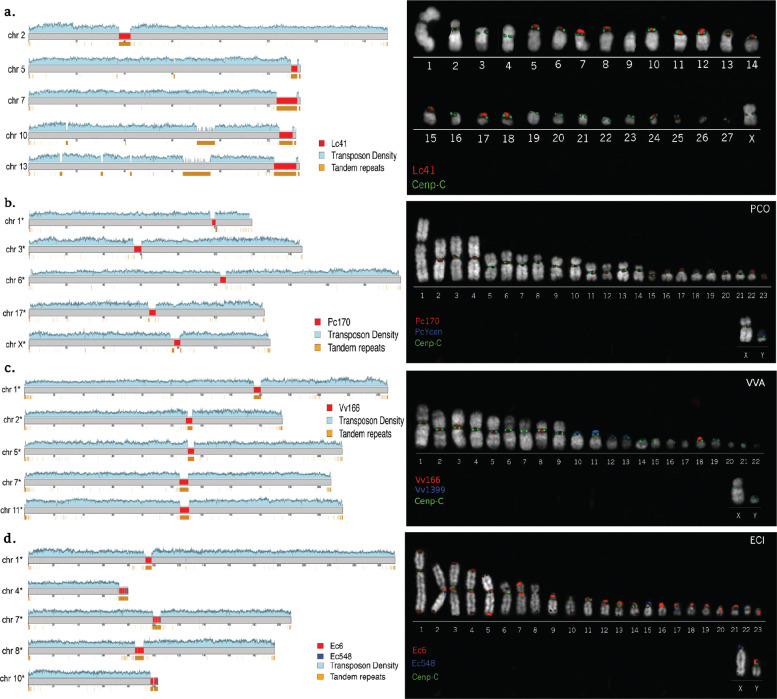
Centromere sequence validation by FISH. We depict five chromosome contig sequences (left) as ideograms of four lemur species: a. LCA, b. PCO, c. VVA, and d. ECO based on sequence and assembly. Centromeric (red) and pericentromeric repeats (blue for VVA and ECO) are shown with respect to transposon density, which drastically reduces at the putative centromeres. Co-immunohybridization (right panels) experiments showing FISH with candidate sequence probes (red) and CENP-C antibody signal (green) validate centromeric localization (closely related ECI is used in lieu of ECO). All chromosomes with an asterisk (*) are named according to their synteny with respect to LCA.

In order to validate our candidates, we performed FISH (fluorescence in situ hybridization) and immunoFISH experiments for strepsirrhine species for which we had access to available cell lines for experimental testing (n=4). For example, we superimposed our centromere candidate sequences for *Lemur catta* (LCA) onto chromosomal scaffolds developed as part of the NCBI reference (mLemCat1, GCF_020740605.2). Similar to haplorhines, we observe a notable reduction in transposon/retrotransposon density corresponding to the long tandem repeat arrays (Figure 3). To confirm that these repeat arrays define centromeres, we constructed fluorescently labelled DNA probes specifically based on the LCA monomer for FISH imaging. Probes constructed with 41 bp monomers (repeat Lc41) were hybridized to metaphase spreads generated from LCA cell lines simultaneously labelled with CENP-C monoclonal antibodies. These immunoFISH experiments reveal that CENP-C antibodies and Lc41 FISH probes co-localize with the position of the primary constriction defining the centromeres. As expected, based on the telocentric lemur karyotype (Chu and Swomley, 1961), we find the centromere and Lc41 tandem repeat arrays located at the ends of chromosomes depicted in the karyotype (Figure 3a). Of interest, we failed to identify centromeres for four LCA chromosomes, including chromosome X. We presume that these chromosomes may have a minuscule centromere or potentially consist of non-repetitive neocentromeric DNA (Ventura et al., 2001; Cappelletti et al., 2025), and thus, would not have been detected by our approach.

We repeated this experimental validation approach for three additional lemur species (PCO, VVR and a *Eulemur* sp.) where cell lines could be obtained (courtesy of Christian Roos), performing co-immunoprecipitation experiments with CENPC antibodies and FISH DNA-probes designed to the predominant centromeric repeat identified from genome sequence and assembly. In all cases, centromeres or pericentromeric signals were confirmed (Figure 3b,c). Due to lack of an available cell line for *Eulemur collaris*, we used a closely related species instead, the gray-headed lemur (*Eulemur cinereiceps*), which confirms the dual role of telomeric repeats to define both the ends of the chromosome and the centromere associated with kinetochore binding (Figure 3d). See below for more detail.

### Comparative primate centromere and structure analyses.

**Figure 4 |:**
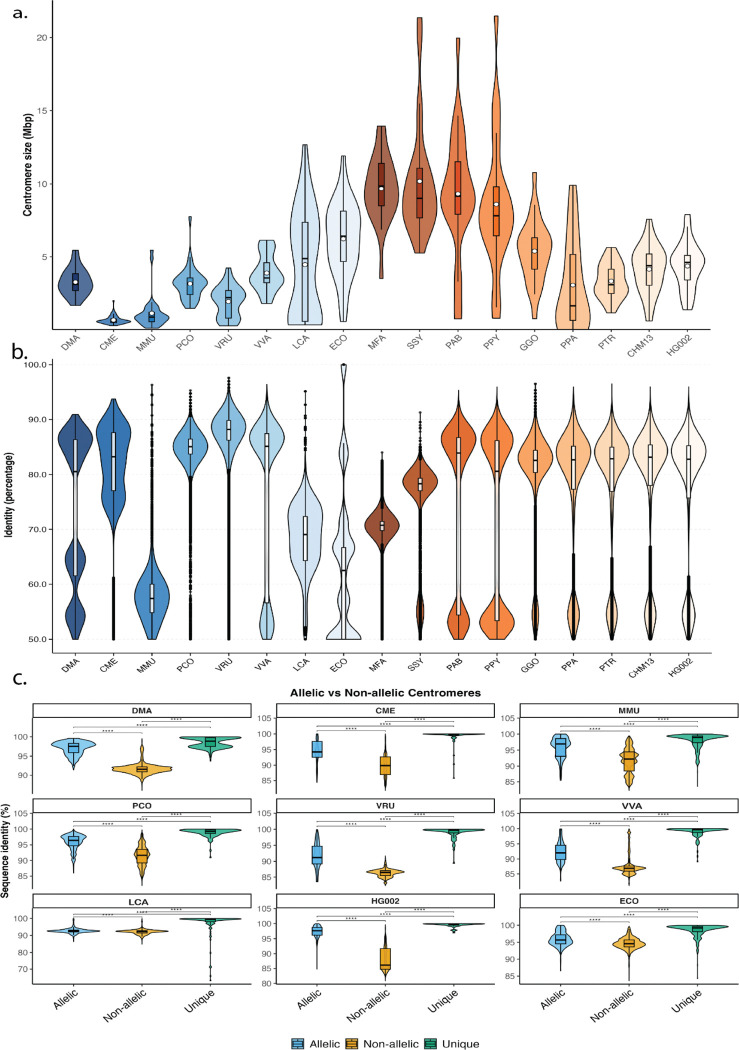
Comparative analyses of primate centromere length and divergence. a, Comparative centromere size distribution from lemurs, MFA and apes, including T2T-CHM13 and the diploid human genome HG002. b, Identity distribution of all the individual centromeric monomer units in a species, with respect to the most frequent monomer in that species. For non-lemur primates, canonical α-satellite was taken as the reference. c, Comparison between the identities of allelic and non-allelic centromeres in each species. Identities of unique sequences in every genome are plotted as control for the genome average.

Overall, we find that centromeres exhibit distinct characteristics among diverse lemur species, with each genus possessing distinct single monomers or even two monomers in the same species. In general, the total average length of HORs appear smaller (typically less than 5 Mbp) in size when compared to eight haplorhine species that were recently T2T sequenced (Yoo et al., 2025; Zhang et al., 2025) (Figure 4). This difference is also statistically significant when we perform centromere group comparisons between strepsirrhine and haplorhine (Figure 4a, Supplementary Figure S4) (p < 2.22e-16, Wilcoxon rank sum test). We also compared monomer sequence divergence with respect to each consensus sequence (Table 2) and contrasted it with each other and canonical alpha-satellite divergence for haplorhines. Most lemur species follow a unimodal distribution: LCA, VRU, PCO, MMU, and CME (Figure 4b). DMA shows a distinct tri-modality in distribution as the centromeric repeats have diverged to give two new shorter “variants” of the 267 bp monomer, with consensus sizes of 222 and 209 bp. VVA shows a bimodal distribution, which points to other interspersed sequences in its centromeres.

**Figure 5 |:**
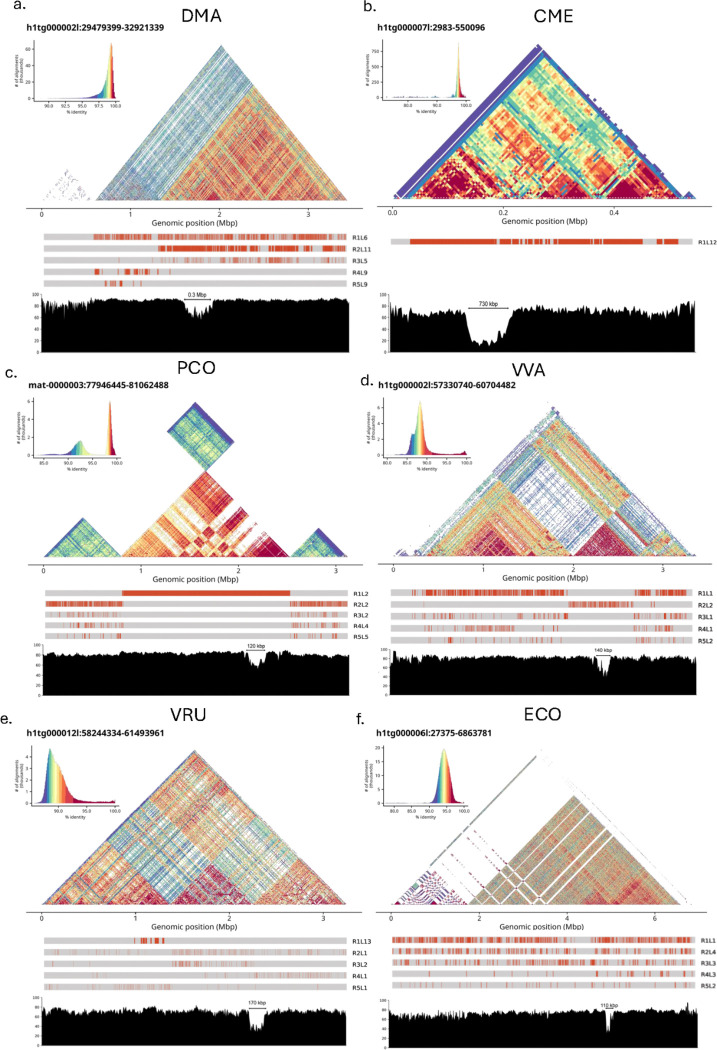
Lemur centromere higher-order repeat (HOR) structure and methylation. Heatmap of a centromere from each of six species (DMA, CME, PCO, VVA, VRU and ECO), along with their HOR annotations and 5-methylcytosine profiles. The HOR annotations show different HOR structures with score rankings as ‘R’ and length of individual HOR as ‘L’. CME has only a single HOR. Hypomethylation centromere dip regions and their estimated length (arrows) are shown in the context of the HOR structure and are variable between species.

Because we typically resolve two homologues for each chromosome, we could also compare the allelic and non-allelic sequence identity of centromeres and contrast it with unique flanking sequences in the assemblies. As expected, across species allelic centromeres share higher sequence identity to each other than to those from other chromosomes with identities ranging from lowest allelic median for VVA at 91.18 ± 3.94% to highest for MMU at 96.94 ± 3.17%. Non-allelic centromere identity medians ranged from 86.43 ± 1.22% again in VVA to 94.59 ± 1.76% in ECO. For each strepsirrhine species, we summarized the HOR structure using StainedGlass (Vollger et al., 2022) and HiCAT (Gao et al., 2023) and use the tool’s standard notation to describe HOR organization (i.e., R1L6 defines the highest-ranked HOR, which maximizes both coverage and repeat fidelity (Methods) while L denotes the length defined by the number of repeating monomer units in an HOR cassette). We also investigate 5-methyl cytosine features of the corresponding heterochromatin associated with each centromere, searching for evidence of a CDR (Figure 5). We summarize these centromere features individually for each species below.

### Aye-aye (DMA) –

We assembled 19 centromeres in DMA, out of 30 total chromosomes. We distinguish three different but inter-related candidate centromeric repeats in DMA, whose sizes are 267 bp (Dm267), 222 bp (Dm222), and 209 bp (Dm209). We find that all the repeats have about 50% global pairwise sequence identity with each other but have a nearly identical core region of 75 bp, which aligns pairwise with greater than 95% identity in all three sequences. When compared to previously described monomers, DMA1 (146 bp) and DMA2 (268 bp) from Lee et al. (2011), DMA2 is identical to Dm267, but DMA1 makes partial sequences of all the other three sequences, along with DMA2 (Supplementary Figure S5a). We find that the centromeres consist of at least two of the three Dm sequences: Dm267 and Dm209, usually tandem repeats of one form and/or alternating with each other (see Supplementary Figure S5b and Supplementary Figure S4c for higher resolution views of the two centromeres). Overall, Dm267, Dm209 and Dm222 account for 43%, 32% and 14% base pair sequence, respectively, in all the complete centromeres. The other ~11% of the centromere contains unique sequences. The most frequent monomer, Dm267, is organized into an R1L6 HOR structure in 15 out of 19 complete centromeres, with different secondary HORs in different centromeres. 5-methyl cytosine analysis shows distinguishable CDR for 17 assembled DMA centromeres, with the other two having small tentative dips. We estimate that CDRs for DMA range from 100 kbp to 300 kbp, with an average of 180 kbp (Figure 5a).

### Dwarf lemur (CME) –

For this species, we recovered the largest number of centromeres, with 64 of 66 chromosomes showing complete centromere assembly. The predominant centromere motif, Cm143, is a highly AT-rich (70.3%) monomer. The centromere structure consists of only one monomer, though of note, we find 11 centromeres that consist of two highly diverged HORs with identity of 72% between both the repeat arrays (Supplementary Figure S7). All the other chromosomes contain a single prominent HOR structure, with R1L3 being the most common (31 centromeres) followed by R1L12 in seven centromeres and another 26 centromeres having varied HOR configuration (as confirmed by CENdetectHOR; Daponte et al., 2025, Supplementary Table 5). The average size of CME centromeres at 520 kbp is significantly smaller than that observed for other lemur species (p < 0.01) (Figure 4a), but proportionally the CDR is larger. For example, the centromere in Figure 5b has a CDR 130 kbp long, which is about 38.4% of the total centromere size. Other 57 centromeres have a single CDR, six have two CDRs each, and one was observed with three CDRs.

### Gray mouse lemur (MMU) –

For this species, the monomer was previously described as Mm53, a 53 bp repeat (Larsen et al., 2017). We find a similar repeat consensus but with one additional base pair, thus relabelling it Mm54. We successfully characterize 28 centromeres from the whole karyotype of 66 chromosomes. There is no prominent HOR structure in MMU centromeres. We find CDRs in five centromeres only, with inconsistent methylation pattern for the remaining other 28 centromeres (Supplementary Figure S13). The basis for this reduced CDR signal for the mouse lemur is unknown. We note, however, that the DNA used for this particular sample was extracted more than 4 years ago and methylation signals are known to degrade over time (Lee et al., 2023).

### Coquerel’s sifaka (PCO) –

Sifaka has a 170 bp consensus centromere motif sequence (Pc170), similar in size to the α-satellite monomer in haplorhines, but there is otherwise no homology. We were able to assemble 33 centromeres out of 48 total chromosomes.The availability of parental data allowed this assembly to be fully phased and we successfully assigned 16 centromeres to the paternal haplotype and 17 to the maternal haplotype. All sequence-resolved centromeres in PCO show a well-defined dimer array: R1L2 is the predominant HOR and the same HOR structure was identified by CENdetectHOR (Daponte et al., 2025), with only three centromeres as exceptions (Supplementary Table 5). HORs are organized around a high sequence identity core, flanked by lower, more divergent flanking satellites. The CDR is very clearly demarcated in the centromeres, also overlapping the aforementioned high-identity region. All the CDRs are consistently between 110–150 kbp in all the centromeres (Figure 5c).

### Black and white ruffed lemur (VVA) –

Candidate assembled centromeres decompose into two monomers of distinct size: 166 bp (Vv166) and a much longer 1399 bp (Vv1399). From the expected karyotype of 46 chromosomes, we assembled 21 candidate centromeres, of which 16 consist solely of Vv166, three only of Vv1399, and the other two comprised of a fusion of both Vv166 and Vv1399 arrays in tandem, or one flanking the other (Supplementary Figure S8). For the centromeres consisting only of Vv166, R1L1 is the most frequent HOR configuration in six centromeres, but there was considerable variation in other HOR configurations found in other centromeres, ranging from R1L3 to R1L35. For the Vv1399 centromere, there is only one HOR, R1L1. While examining heterochromatin methylation, we observe an unexpected CDR depending on the type of centromeric arrays. We note a single canonical CDR for Vv166-based centromeres (Figure 5d), in contrast to multiple narrower CDRs for centromeres composed of Vv1399 or mixed/fused Vv1399/Vv166 (Supplementary Figure S9). This may suggest a sequence dependence on the pattern of CDR formation. Co-ImmunoFISH showed perfect colocalization between the probe Vv166 and CENP-C protein in 9 out of 24 chromosomes, while the probe Vv1399 displayed neither colocalization with immunohybridization signals nor with Vv166 FISH probes. Instead it identified pericentromeric loci for 5 out of 24 Vva chromosomes (Figure 3c). These results suggest a dual composition of *Varecia* centromeres: Vv166-associated functional centromeres, clearly marked by CENP-C colocalization, and centromeres where we were not able to identify satellite components in chromosomes lacking Vv166 signal. Vv1399, instead, is best interpreted as a pericentromeric satellite without detectable association with centromeric function. One possibility may be that the lack of detectable FISH signals for Vv166 for other chromosomes may result from the presence of satellite arrays whose size in these chromosomes is below the resolution of FISH.

### Red ruffed lemur (VRU) –

Given that this congeneric species diverged from VVA recently during the Pleistocene period (dos Reis et al., 2018), the predominant monomers are, unsurprisingly, homologous to VVA. Following the pattern of VVA, VRU centromeres consist of two monomers, which are nearly identical to VVA. For the longer one, the consensus motif has lengthened by 6 bp (Vr1405), while the other, more abundant motif remains the same in length (Vr166). We assembled 20 centromeres in VVA, where 11 are composed almost entirely of Vr166, three are composed of only Vr1405, and six contain both in which small arrays of Vr1405 are flanking the Vr166 array. R1L1 is the most frequent HOR in the Vr166 monomer with four centromeres, with substantial variation of HOR lengths in other centromeres. The methylation patterns also follow the same trend with Vr1405 centromeres having multiple CDRs in vicinity of each other, in contrast to a single CDR for the Vr166 centromeres (Figure 5e).

### Collared brown lemur (ECO) –

We discovered a particularly unexpected centromere composition in this species. Known telomere repeats of ‘TTAGGG’ form long interstitial arrays of average length 6.22 Mbp that define the putative assembled centromeres. This repeat (Ec6), along with sporadically interspersed Ec548 repeats, predominate in all 32 centromeres that we successfully assembled for ECO (2n ~ 50). These interspersed filler regions are readily apparent in the StainedGlass heatmaps (Figure 5f). Due to the very small size of the repeat monomer, we could not distinguish HOR structures for centromeres except one that showed the R1L1 structure according to HiCAT, which implies that it does not contain any HOR structure. Nevertheless, we identify at least one CDR in every centromere, and for six centromeres we observe two CDRs. Co-immunoFISH experiments confirm that the telomeric repeat sequence has been co-opted to function as a centromere with typical kinetochore binding properties, while the satellite Ec548 appears to label pericentromeric satellite DNA, rather than as a sequence directly associated with centromeric activity (Figure 3d). The reuse of a telomeric repeat to define both the telomere and centromere within *Eulemur* may contribute to the high levels of variability in karyotype numbers seen in general within the brown lemur clade (Rumpler and Dutrillaux, 1976; Ventura et al., 2001).

### Ring-tailed lemur (LCA) –

For LCA, we scaffolded our assembly, using RagTag (Alonge et al., 2022), on the whole-karyotype assembly of 56 chromosomes from NCBI (GCF_020740605.2). We recovered 25 assembled centromeres where a 41 bp monomer (Lc41) predominates. We also identify other monomers extending the basic 41-mer to 76 bp and 117 bp. Due to this variation and interdigitation of non-canonical derivatives of Lc41 including smaller motifs, LCA centromeres show comparatively less sequence identity when compared to other LCA centromeres (Supplementary Figure S10). We did not find any prominent HOR structure in the LCA centromeres. Unlike most other lemur centromeres, we found no evidence of a clearly defined CDR for any centromere; instead, methylation patterns continuously fluctuate (Supplementary Figure S11). Co-immunoprecipitation experiments, however, confirm Lc41 as the centromere repeat motif (Figure 3a).

### Strepsirrhine CENP protein evolution.

To assess whether centromere-associated proteins show signatures of adaptive evolution specific to lemurs, we performed clade-specific selection analyses on codon-aligned sequence for three genes encoding proteins critical for kinetochore function: CENP-A, CENP-B, and CENP-C (Methods). We partitioned the species tree into catarrhines, platyrrhines, and lemurs and treated each clade in turn as the “foreground.” Signals are strongest with CENP-B. With lemurs as “foreground,” PAML detects positive selection at approximately 9% of sites of CENP-B (ω = 3.00, p < 0.0001), against a backdrop of strong conservation across other primates (global ω = 0.10). Multiple independent tests corroborate this finding for CENP-B: RELAX indicates that positive selection strength on CENP-B is nearly twice as intense in lemurs when compared to other primates (K = 1.86, p = 0.0001) and BUSTED confirms gene-wide positive selection on lemur branches (p = 0.010). Importantly, neither catarrhines nor platyrrhines show comparable signals when tested as foreground, establishing that this is a lemur-specific phenomenon rather than a general primate pattern. Moreover, CENP-A shows site-specific positive selection in both lemurs (ω = 4.49, p = 0.003) and catarrhines (ω = 5.99, p = 0.021), with RELAX and BUSTED yielding non-significant signals. CENP-C shows a significant lemur signal in PAML and BUSTED (p = 0.001 and p = 0.005) but the RELAX result is not significant. Together, these results indicate strong positive selection on *CENPB* specific to lemurs, with weaker, less consistent signals for *CENPA* and *CENPC*.

## DISCUSSION

Centromeres harbor some of the most rapidly evolving repetitive sequences in eukaryotic genomes, and among primates most of their characterization has been restricted to the Old World Monkey lineages (Csink and Henikoff, 1998; Logsdon et al., 2024). We apply long-read sequencing methods to eight lemur species to investigate centromere evolution, repositioning, and chromosomal structural variation at an unprecedented resolution. The haplorhine lineage, comprising Platyrrhini, Catarrhini, and Tarsiidae, whose common ancestor lived ~67–75 million years ago (dos Reis et al., 2018), is unified by α-satellite DNA as the predominant centromeric repeat, with variation observed among HOR organization between apes, Old World and New World monkeys (Yoo et al., 2025; Cacheux et al., 2018; Zhang et al., 2025; Nishihara et al., 2021). In contrast, lemurs exhibit a fundamentally different centromeric landscape: we identify seven distinct satellite monomer families across eight species spanning ~41 million years, with no motif shared across distinct genera. This implies turnover of the predominant centromeric repeat at ~1.7 motif changes per 10 million years, far exceeding haplorhine conservation (1 motif/~40 million years). This is consistent with more rapid centromere evolution, potentially as a result of an ongoing centromere drive arms race. Whether centromeric repeats are a cause or consequence of centromere evolution remains debated, with evidence supporting a meiotic drive-mediated competition and feedback loop between satellite DNA and histone proteins, an evolutionary arms race underlying the centromere paradox (Henikoff et al., 2001; Cooper and Henikoff, 2004; Logsdon et al., 2020).

In our study, the convergence of a positive selection model on CENP-A and CENP-B in lemurs is consistent with the centromere drive model and provides the clearest molecular evidence yet for an ongoing arms race. Moreover, the signal for CENP-B emerges against a background of strong purifying selection, consistent with the broad mammalian conservation of CENP-B reported in previous studies (Sullivan and Glass, 1991; Okada et al., 2007), making its lemur-specific departure all the more compelling. This has direct relevance to the proposed role of centromere incompatibility in lemur speciation: if CENP-B was evolving adaptively in the ancestors of extant lemur clades, centromere recognition machinery was diverging concurrently with the karyotypic changes, providing a molecular mechanism through which centromere drive could have contributed to hybrid meiotic dysfunction and reproductive isolation.

We contend that this arms race, centromere repositioning, and overall chromosomal evolution have had a major role in the diversification of lemur species and may be consistent with White’s model of stasipatric speciation (White et al., 1968; Kearney and Hewitt, 2009). White championed the idea of stasipatric speciation in his writings, but it remained a fringe theory due to lack of data explaining fixation and spread of chromosomal variants (White, 1968; White, 1978; King, 1993). High species-specific centromere turnover rates from this study as well as previous observations of highly variable karyotype numbers and multiple fissions and fusions between closely related lemur species may all be linked (Rumpler and Albignac, 1975; Kolnicki, 2000; Rocchi et al., 2012). There is ample evidence that chromosomal rearrangements suppress recombination from studies in apes (Lin et al., 2025; Porubsky et al., 2020), deer mice (Hager et al., 2022), wild mice (Marin-Garcia et al., 2024), and muntjacs (Yin et al., 2021), consistent with White’s stasipatric model. Meiotic drive is considered to be the mechanistic basis of selecting favored states of karyotypes, mainly either predominantly acrocentric or metacentric (Pardo-Manuel de Villena and Sapienza, 2001; Blackmon et al., 2019). We hypothesize that meiotic drive may be a core mechanism in lemur speciation, ensuring that particular variants of centromeres get selected particularly during female gametogenesis and become fixed in the parent population along with the chromosomes that carry them. Meiosis in females is asymmetric and can select for the particular variants that favor the egg over the polar bodies. Later, these divergent centromere monomers in different populations would cause differential CENP protein loading and meiotic dysfunction in hybrids, resulting in reproductive isolation despite all incipient lemur species residing within the same geographic island.

**Figure 6 |:**
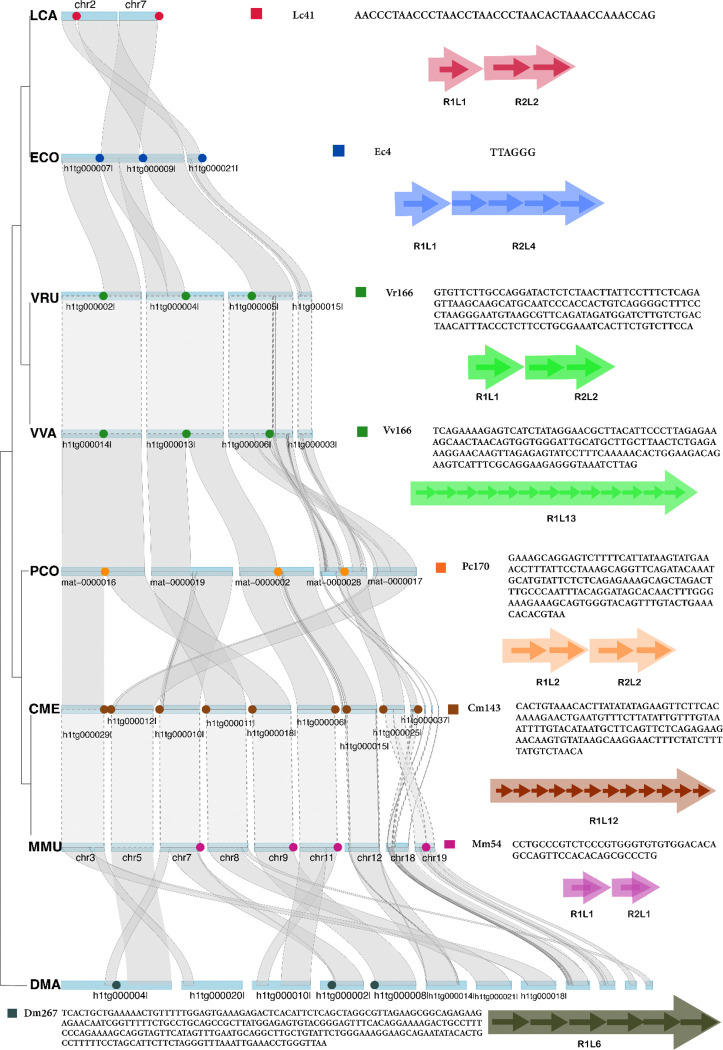
Centromere composition and repositioning with respect to synteny breakpoints among the lemurs. Mapping of two LCA chromosomes across all the related species showing the repositioning of centromeres and breakpoints of synteny across the lemur clade. Also described are the monomer sequences of each species and their most frequent HOR structure of each species. Dashed synteny lines across two species pairs demonstrate a near perfect synteny conserved between the phylogenetically closely related species.

We further suggest that this process is concomitant with formation of evolutionary new centromeres, a process previously indicated by FISH studies on *Lemur catta* and *Eulemur fulvus* X chromosomes (Ventura et al., 2004; Rocchi et al., 2012), which should also have accentuated lemur diversification. Lemur karyotypes provide direct empirical support for this model. They have a preponderance of acrocentric chromosomes (Rumpler and Dutrillaux, 1976; Horvath and Willard, 2007), and a putative role of Robertsonian translocations in lemur evolution has been suggested since their first karyotypic characterizations (Chu and Swomley, 1961). This is now confirmed at sequence resolution (Figure 6), where syntenic blocks have broken across all species at centromere junctions, providing evidence of ancestral inter-specific Robertsonian translocations and centromere repositioning. Across all seven genera, centromeres have shifted from their syntenically conserved positions, except in both *Varecia* species, where sequence and positional conservation are maintained, and in Cheirogaleidae, where only synteny is conserved—consistent with their comparatively lower rates of karyotypic diversification.

Evolutionary new centromere formation is most strikingly observed in the *Eulemur collaris* (ECO) genome, where telomeric repeats appear to be functioning as centromeric repeats, demonstrating recent chromosome rearrangement events. The *Eulemur* genus is known to have a varied karyotype as well as high rates of speciation (Ventura et al., 2001), and these results strengthen the case that telomeric repeats play a prominent role in centromere and karyotype evolution more broadly (Villasante et al., 2007). The presence of telomeric repeat sequences at both the centromeres and the ends of chromosomes may relate to the high levels of variability in karyotype numbers reported in general for the brown lemur clade (Rumpler and Dutrillaux, 1976; Ventura et al., 2001).

This is not to say that allopatric speciation also did not play a significant role in lemur diversification. Past geographical events, especially during the Oligocene and Miocene periods, likely contributed to separating populations (Antonelli et al., 2022). Additionally, the emergence and expansion of new ecological niches—including grasslands alongside dry and humid forests, with rivers acting as further barriers—may have promoted lemur speciation and the overall biodiversity of the island (Goodman and Ganzhorn, 2004; Igea and Tanentzap, 2021; Razafindratsima et al., 2025). We posit that, alongside these abiotic factors, molecular mechanisms such as centromere repositioning and chromosomal rearrangements provided speciation plasticity among phylogenetically related incipient species in close geographic proximity. The remarkable karyotypic diversity observed across lemur genera, and the species-specific centromere monomers and their rapid evolutionary turnover, are consistent with repeated shifts in the direction of meiotic drive, making lemurs an exceptional natural system in which to observe this process at genomic resolution. Linking chromosomal rearrangements and centromere evolution to macroevolutionary processes like speciation is a challenge, and empirical experiments with higher animals are simply not feasible (Harvey et al., 2019). These eight genome assemblies, however, open the door to functional studies of CENP binding divergence across species, hybrid fitness predictions from sequence data alone, and a comparative framework for testing centromere-driven speciation.

## Supplementary Material

Supplement 1

## Figures and Tables

**Table 1 –:** Summary of sequence and assembly statistics

	*Microcebus murinus (MMU)*		*Cheirogaleus medius (CME)*		*Propithecus coquereli (PCO)*		*Daubentonia madagascariensis (DMA)*		*Lemur catta (LCA)*		*Varecia rubra (VRU)*		*Varecia variegata (VVA)*		*Eulemur collaris (ECO)*	
	Hap1	Hap2	Hap1	Hap2	Hap1	Hap2	Hap1	Hap2	Hap1	Hap2	Hap1	Hap2	Hap1	Hap2	Hap1	Hap2
***Bases (Gbp)***	2.383	2.351	2.25	2.307	2.382	2.276	2.505	2.431	2.299	2.187	2.229	2.146	2.262	2.144	2.261	2.213
***Contigs***	241	125	69	45	301	425	113	86	127	188	46	46	58	52	84	60
***N50 (Mbp)***	93.72	104.15	102.37	97.27	119.49	129.53	112.88	116.68	112.59	109.01	174.2	174.75	174.77	151.14	129.82	105.83
***100k+ (Gbp)***	2.377	2.349	2.249	2.307	2.371	2.263	2.503	2.431	2.295	2.181	2.229	2.146	2.262	2.144	2.26	2.213
***Near T2T contigs***	8	3	20	16	7	8	2	2	13	13	8	6	7	3	15	14
***Contigs with N (undetermined)***	27	24	0	0	8	8	0	0	4	3	0	0	0	0	0	0
***Read Coverage (HiFi/ONT)***	42.62/45		102.86/67.15		87.54/128.62		97.51/55.99		73.79/42.58		89.91/26.97		90.16/26.33		93.54/51.40	

**Table 2 -:** Summary of the assembled centromeres

Species	Centromere motif	Number of assembled centromeres (both haps)	Length	GC content	Most frequent HOR length
**Aye-aye (DMA)**	Dm267	19	267	43.82	6
	Dm209		209	40.67	
	Dm222		222	39.64	
**Dwarf Lemur (CME)**	Cm143	64	143	28.67	3
**Mouse lemur (MMU)**	Mm54	28	53	67.92	-
**Coquerel’s sifaka (PCO)**	Pc170	33	171	39.05	2
**Ring-tailed lemur (LCA)**	Lc41	25	41	43.9	-
**Red ruffed lemur (VRU)**	Vr166	20 (11 of only Vr166, 3 only Vr1405)	166	42.17	1
	Vr1405		1405	46.05	
**Black and white ruffed lemur (VVA)**	Vv166	21 (16 of only Vv166, 3 of only Vv1399)	166	42.17	1
	Vv1399		1399	45.46	
**Collared brown lemur (ECO)**	Ec6	32	6	50	-
	Ec548		548	47.08	

**Table 3 -:** Tests of selection on CENP genes

Gene	foreground	M0 ω	LRT M1aM2a Significance	LRTM7M8 Significance	BS Significance	BS (fg) ω	BS proportion
**CENPA**	Lemur	0.5003	*	**	**	4.4929	0.0445
**CENPA**	OWM	0.5003	*	**	*	5.9905	0.03
**CENPA**	NWM	0.5003	*	**	ns	2.5081	0
							
**CENPB**	Lemur	0.1041	ns	***	***	3.0049	0.0886
**CENPB**	OWM	0.1041	ns	***	ns	11.3848	0.0014
**CENPB**	NWM	0.1041	ns	***	ns	1	0
							
**CENPC**	Lemur	0.7057	***	***	**	2.8099	0.0364
**CENPC**	OWM	0.7057	***	***	ns	1.6704	0.044
**CENPC**	NWM	0.7057	***	***	ns	4.8556	0.0055
							
**TRIM5**	Lemur	1.1519	***	***	***	6.1327	0.0364
**TRIM5**	OWM	1.1519	***	***	***	8.9436	0.0177
**TRIM5**	NWM	1.1519	***	***	***	8.2967	0.0312
							
**LYZ**	Lemur	0.5877	***	***	***	8.4768	0.0245
**LYZ**	OWM	0.5877	***	***	ns	2.6782	0.0999
**LYZ**	NWM	0.5877	***	***	ns	4.5516	0.0197

(ω - dN/dS ratio, LRT - likelihood ratio test, BS - branchsite, ns - not significant, * p < 0.05, ** p < 0.01, *** p < 0.001)

## Data Availability

The raw genome sequencing data and assembly data generated for this project are available from Genbank with BioProject identifiers PRJNA1459402 - PRJNA1459415 and accession numbers SAMN58406792 - SAMN58406798. The gray mouse lemur (*Microcebus murinus*, MMU) genome is available under the accessions: GCA_040939455.2 and GCA_040939475.2. The assembly evaluation pipeline is available on (https://github.com/EichlerLab/assembly_eval) and (https://github.com/EichlerLab/assembly_qc). Code for centromere identification and selection analysis is available on (https://github.com/trihim/lemur_centromere).
