# Supplementary material for: Rapid centromere turnover and the adaptive radiation of lemurs
Source: bioRxiv. 2026 May 19:2026.05.16.725662. Preprint. [Version 1] doi: 10.64898/2026.05.16.725662 (PMC13228423; doi:10.64898/2026.05.16.725662)
Supplement: Supplement 1 [file NIHPP2026.05.16.725662v1-supplement-1.pdf]

bioRxiv preprint doi: https://doi.org/10.64898/2026.05.16.725662; this version posted May 19, 2026. The copyright holder for this preprint (which was not certified by peer review) is the author/funder, who has granted bioRxiv a license to display the preprint in perpetuity. It is made available under a CC-BY 4.0 International license.

Supplementary Material

bioRxiv preprint doi: https://doi.org/10.64898/2026.05.16.725662; this version posted May 19, 2026. The copyright holder for this preprint (which was not certified by peer review) is the author/funder, who has granted bioRxiv a license to display the preprint in perpetuity. It is made available under a CC-BY 4.0 International license.

Table of Contents

Methods

A. Genome Assembly
B. Phylogenetic Reconstruction and Whole-Genome Alignment
C. Centromere Identification and Characterization
D. Molecular Evolution of Centromere Proteins

Figures

S1. Improvement in assemblies' contiguity
S2. Synteny of *Lemur catta* genome with Macaca fascicularis (MFA)
S3. Breakpoints of lemur genome with respect to MFA
S4. Centromere size comparison in strepsirrhines and haplorhines
S5. Detailed features of *Daubentonia madagascariensis* centromere
S6. StainedGlass heatmaps for all assembled centromeres of *Daubentonia madagascariensis*
S7. StainedGlass heatmaps for all the centromeres of *Cheirogaleus medius*
S8. Ideogram showing centromeres on contigs of *Varecia variegata* (VVA)
S9. Methylation profiles of VVA centromeres consisting of different monomers
S10. StainedGlass heatmaps for all the centromeres of *Lemur catta*
S11. Structure of a single *Lemur catta* centromeres, showing absence of a prominent CDR
S12. Structure of a single *Microcebus murinus* centromere
S13. StainedGlass heatmaps for all *Microcebus murinus* centromeres
S14. StainedGlass heatmaps for all *Propithecus coquereli* centromeres
S15. StainedGlass heatmaps for all *Varecia variegata* centromeres
S16. StainedGlass heatmaps for all *Varecia rubra* centromeres
S17. StainedGlass heatmaps for all *Eulemur collaris* centromeres
S18. Logos plots for all the monomers across eight lemur species
S19. Selection of CENP-B in primates
S20. Raw FISH images of four species

References

bioRxiv preprint doi: https://doi.org/10.64898/2026.05.16.725662; this version posted May 19, 2026. The copyright holder for this preprint (which was not certified by peer review) is the author/funder, who has granted bioRxiv a license to display the preprint in perpetuity. It is made available under a CC-BY 4.0 International license.

Methods

A. Genome Assembly

1. Lemur DNA sequencing and genome assembly. Blood samples were collected from seven lemur species (all except *M. murinus*) at the Duke Lemur Center (Duke IACUC protocols A208-23-10, A010-25-02; DLC protocol BSM-11-24-4) and used for DNA extraction. High molecular weight gDNA was extracted from frozen blood aliquots using the NEB Monarch HMW DNA extraction kit for Cells & Blood (#T3050L) following the manufacturer's protocol. All individuals were sequenced using both PacBio HiFi long reads and Oxford Nanopore Technologies (ONT) ultra-long reads. Haplotype-resolved assemblies were generated using hifiasm v0.19.9 with HiFi and ONT reads for six species. For two species with available parental Illumina data (*P. coquereli* and *L. catta*), assemblies were independently generated with both hifiasm v0.19.9 and Verkko v2.2.1; Verkko assemblies were selected for downstream analyses on the basis of superior assembly contiguity and base-level accuracy. All assemblies were processed through a standardized quality-control pipeline (https://github.com/EichlerLab/assembly_qc). Assembly completeness and error profiles were evaluated using NucFreq (https://github.com/EichlerLab/assembly_eval) and Flagger v0.3.2 (Vollger et al., 2019; Liao et al., 2023). Collapsed regions were defined as loci where the second most frequent base exceeded 5× read depth, indicative of heterozygous sequence collapsed into a single haplotype. Duplicated and HiFi-depleted regions were defined as intervals with absent or markedly reduced HiFi read coverage, consistent with false duplication or assembly dropout, respectively. Assembly quality metrics for all eight species are provided in Supplementary Table S2. For *Lemur catta*, we used RagTag (Alonge et al., 2022) to scaffold the assembly on the reference genome in NCBI – GCA_020740605.1. The chromosomes in our assembly now follow the numbering from Cardone et al., 2002, which were mapped according to the synteny with human T2T-CHM13 genome.

A phased, haploid mouse lemur genome assembly was generated from primary fibroblasts of an adult index female (Chang, Lalgudi, Yoo, Trivedi et al., in preparation). Briefly, genomic DNA was extracted from the index and both parents. The index was sequenced using HiFi, ONT, and Hi-C Illumina platforms, while parental genomes were sequenced using Illumina short reads. A phased scaffold-level assembly was produced with hifiasm (v0.23.0) using trio-binning and ONT integration, followed by Hi-C scaffolding with HapHiC (v1.0.7) to generate a chromosome-level assembly. Assembly quality was evaluated with NucFreq and Flagger per above.

B. Phylogenetic Reconstruction and Whole-Genome Alignment

A distance-based phylogeny was constructed using Mashtree with 100 bootstrap replicates. Sketches were generated with an assumed genome size of 2 Gbp and a minimum k-mer depth of zero to retain all k-mers, using the command:

bioRxiv preprint doi: https://doi.org/10.64898/2026.05.16.725662; this version posted May 19, 2026. The copyright holder for this preprint (which was not certified by peer review) is the author/funder, who has granted bioRxiv a license to display the preprint in perpetuity. It is made available under a CC-BY 4.0 International license.

```
mashtree_bootstrap.pl --reps 100 --numcpus 12 --outmatrix
--genomesize 2000000000 $(<fasta.fofn) -- --min-depth 0 >
mashtree.tre
```

Each lemur assembly was aligned pairwise against two reference genomes—*Macaca fascicularis* (MFA) and the human T2T assembly (T2T-CHM13)—using AnchorWave. Gene annotations for all species were derived from BUSCO gene models produced by compleasm (Huang and Li, 2023) as part of the assembly QC pipeline described above and used as anchor points for the AnchorWave alignment. Final alignments were produced using the `anchorwave proali` command, generating output in MAF format. MAF files were subsequently converted to PAF format using wgatools (Wei et al., 2025) for compatibility with downstream synteny and breakpoint analyses. Synteny breakpoints were identified using an in-house script. Synteny blocks were defined with a maximum intra-block gap in the target sequence of 100 kbp, a minimum query alignment length of 1 Mbp, and a minimum individual alignment length of 10 kbp.

C. Centromere Identification and Characterization

Centromere monomer identification

Centromeric tandem repeat arrays were identified using TRF (Benson, 1999) applied to all eight assemblies. For a subset of species, results were independently corroborated using TRASH (Wlodzimierz et al., 2023), which performs an equivalent analysis and generates analogous summary outputs. This approach follows the framework first established by Melters et al. (2013) for mammalian centromeric repeat identification and has previously been applied in identification of mouse lemur centromeres (Larsen et al., 2017). For each species, repeat consensus length was plotted against total array size. Centromeric repeat arrays were identified by visual inspection of these plots, with centromeric candidates expected to produce peaks at a fundamental repeat unit length and its integer multiples, reflecting the hierarchical structure of satellite DNA. The fundamental repeat monomer for each species was defined as the lowest-order multiple identified from the TRF plots. All sequences of that unit length were extracted from the genome and the most frequently occurring sequence was designated the species monomer. Each monomer was then used as a BLAST query against the full genome assembly to precisely localize centromeric repeat arrays. Where multiple overlapping or adjacent arrays were detected, the longest contiguous array was designated the centromere locus. Monomer sequences were independently validated using MEME (Bailey et al., 2015), with the monomer length explicitly provided as a constraint and the BLAST-defined centromere array as input sequence; results were concordant across all species. These candidate repeat arrays were projected onto chromosomal ideograms to assess genomic distribution. Centromeric identity of candidate arrays was confirmed by fluorescence in situ hybridization (FISH) using probes derived from the identified repeats, co-stained with a CENP-C antibody.

bioRxiv preprint doi: https://doi.org/10.64898/2026.05.16.725662; this version posted May 19, 2026. The copyright holder for this preprint (which was not certified by peer review) is the author/funder, who has granted bioRxiv a license to display the preprint in perpetuity. It is made available under a CC-BY 4.0 International license.

FISH validation of centromeric repeats

Primer design and PCR amplification of centromeric monomers

To generate species-specific probes for the putative centromeric repeats of *Lemur catta* (LCA), *Propithecus coquereli* (PCO), *Varecia Variegata* (VVA) and *Eulemur collaris* (ECO), custom primers were designed based on their respective monomeric sequences to specifically amplify these regions. The primer sequences and the probes size are listed in Supplementary Table 4.

Contrastingly, we targeted the *Eulemur collaris* (ECO) 6 bp monomer, Ec6 (5'-TTAGGG-3'), using a pre-synthesized PNA-Cy3 probe from PNA Bio, Inc.

Genomic DNA was extracted from LCA, PCO, VVA, and ECI fibroblasts cultured in RPMI medium supplemented with 16% Fetal Bovine Serum (FBS), 1% L-Glutamine, and 1% Penicillin-Streptomycin. DNA was isolated using the QIAamp DNA Blood Mini Kit (Qiagen) according to the manufacturer's protocol, quantified using a Nanodrop spectrophotometer (Thermo Fisher Scientific), and stored at –20°C.

PCR amplifications were performed using Thermo Scientific DreamTaq PCR Kit in 25 µL reactions containing 1× Master Mix, 0.25 µM of each primer, and 5 ng genomic DNA. For each reaction, thermal cycling included an initial denaturation at 95°C for 3 min, followed by 35 cycles of 95°C for 30 s, annealing at 55–60°C (depending on the species; see Supplementary Table 3) for 30 s, and 72°C for 1 min, with a final extension at 72°C for 7 min. PCR products were verified by electrophoresis on a 1% agarose gel, confirming the expected sizes.

Probe labeling and hybridization

Each PCR product was fluorescently labeled by a single-cycle PCR incorporating Cy3-dUTP, Fluorescein-dUTP, or Cy5-dUTP depending on the experiment. The reaction was performed in 25 µL containing 1× Taq buffer, 2 mM $MgCl_2$, 0.2 µM primers, 0.2 mM dNTPs, 0.1 mM labeled-dUTP, 0.4% BSA, and 1.25 U recombinant Taq polymerase (recombinant) (Thermo Fisher Scientific, Molecular Biology Grade), using 1 µL of PCR product as template. For each reaction, thermal cycling included an initial denaturation at 94°C for 30 sec, followed by 1 cycle of 94°C for 30 s, annealing at 55–60°C (depending on the species; see Supplementary Table 3) for 30 s, and 72°C for 1 min, with a final extension at 72°C for 10 min.

FISH experiments were performed on metaphase spreads of LCA, PCO, VVA, and ECI. Each cell line was arrested in metaphase with colcemid, incubated in hypotonic KCl (0.56%) for 30 min, pre-fixed with methanol: acetic acid (3:1), centrifuged, and fixed again in methanol: acetic acid (3:1).

Chromosome spreads were dropped onto glass slides, aged at 90°C for 1.5 h and treated with 0.005% pepsin in 0.01 M HCl, followed by three washes, 5 minutes each, in 1× PBS, 0.5 M $MgCl_2$, 4% paraformaldehyde, and a cold-ethanol series (70%, 90%, and 100%).

bioRxiv preprint doi: https://doi.org/10.64898/2026.05.16.725662; this version posted May 19, 2026. The copyright holder for this preprint (which was not certified by peer review) is the author/funder, who has granted bioRxiv a license to display the preprint in perpetuity. It is made available under a CC-BY 4.0 International license.

Monomer-based probes were fluorescently labeled according to the target species:

- For *Lemur catta* (LCA), the Lc41 monomer probe was labeled with Cy3.
- For *Varecia variegata* (VVA), the Vv166 monomer and the Vv1405 probes were labeled with Cy3 and Fluorescein (Fx), respectively.
- In *Propithecus coquereli* (PCO), the Pc170 probe was labeled with Cy3 and the probe targeting the Y centromere with Fx.
- For *Eulemur cinereiceps* (ECI), the Ec6 monomer was targeted using the PNA-Cy3 probe, while the Ec548 monomer-base probe with Fx.

All probes were subsequently precipitated by ion-exchange alcohol precipitation without Cot DNA (being probes targeting repetitive regions). Pellets were air-dried and resuspended in a hybridization buffer (50% formamide, 10% dextran sulfate, 2X saline sodium citrate (SSC)). For single-probe FISH, each probe was hybridized independently. For co-hybridization experiments, the two probes were co-precipitated and applied simultaneously to the same metaphase preparations.

For most species, hybridization was carried out overnight at 37°C after denaturation for 2 min at 70°C in HYBrite™ Vysis. The only exception was *Eulemur cinereiceps*; due to the requirements of the PNA probe, a modified protocol was applied with denaturation performed at 75°C for 10 min. Post-hybridization washes were performed at 60°C in 0.1× SSC (three times, 5 min each), with the exception of ECI where washes were carried out at 60°C in 2x SSC, 0.1% Tween (two times, 5 min each). At the end, the slides were stained with DAPI and covered with coverslip, and signals were detected with specific filters using a Leica DMRXA epifluorescence microscope equipped with a cooled CCD camera (Princeton Instruments). Finally, images were processed using Adobe Photoshop™ software.

Immuno-FISH with Cenp-C antibody

LCA, PCO, VVA, ECI cell lines were colcemid-treated and incubated in hypotonic KCl (0.56%) for 30 min, pre-fixed with methanol:acetic acid (3:1), centrifuged, and fixed in methanol:acetic acid (3:1). Chromosome spreads were dropped onto glass slides and incubated for 4 days at 37°C.

Slides were rehydrated in 1× PBS-azide buffer (10 mM NaPO4, pH 7.4, 0.15 M NaCl, 1 mM EGTA, and 0.01% NaN3) for 15 min at RT and washed three times in 1× TWEEN buffer containing 0.5% Triton X-100 and 0.1% BSA.

Mouse anti-CENP-C (Abcam, ab50974) monoclonal antibodies were diluted to 0.001 µg/µL and incubated on the slides (100 µL/slide) for 2 h at 37°C. Slides were washed three times in 1x KB buffer (10 mM Tris-HCl, pH 7.7, 0.15 M NaCl, and 0.1% BSA) and incubated with 100 µL/slide goat anti-mouse IgG secondary antibody conjugated to fluorescein (Fx) (Abcam, ab6785; 1:100 dilution) for 45 min at 37°C in the dark. After incubation with the secondary antibody, slides were washed three times with 1× KB for 2, 5, and 3 min, prefixed in 4% paraformaldehyde in 1× KB for 45 min at RT, and then fixed in methanol: acetic acid (3:1) for 15 min.

bioRxiv preprint doi: https://doi.org/10.64898/2026.05.16.725662; this version posted May 19, 2026. The copyright holder for this preprint (which was not certified by peer review) is the author/funder, who has granted bioRxiv a license to display the preprint in perpetuity. It is made available under a CC-BY 4.0 International license.

For each cell line, FISH was then performed using the species-specific monomer-based probes differently labeled.

For single-probe FISH, each probe was hybridized independently. For co-hybridization experiments, the two probes were co-precipitated, as previously described, and applied simultaneously to the same metaphase preparations after resuspension in hybridization buffer (50% formamide, 10% dextran sulfate, 2× SSC). Hybridization was carried out overnight at 37°C after denaturation for 8 min at 70°C in HYBrite™ Vysis. Post-hybridization washes were performed at 60°C in 0.1× SSC (three times for 5 min, high stringency). Slides were counterstained with DAPI and imaged with a Leica DMRXA2 epifluorescence microscope. DAPI, Cy3, fluorescein, and Cy5 signals were acquired separately with dedicated filters, recorded as grayscale images, pseudocolored, and merged using Adobe Photoshop™.

Methylation and higher-order repeat (HOR) organization

ONT reads for all eight species were basecalled with Guppy 6.5.7, Dorado v1.0.2, or higher with CpG methylation calling enabled. We aligned all the ONT reads to assembled genome and then methylation profiles across centromere arrays were visualized to identify centromere dip regions (CDRs), defined as localized hypomethylated intervals within otherwise highly methylated satellite arrays that mark the site of kinetochore assembly. Profiles were plotted using CDR-finder (Mastrorosa et al., 2024) and CDRs, where present, were manually identified and annotated.

HOR structure was characterized for all assembled centromeres using HiCAT (Gao et al., 2023). HiCAT defines HOR units in the format R*n*L*m*: where the rank (R) is derived from a composite score that integrates centromere coverage with a penalty for over-compressed local nesting, ensuring that the highest-ranked HOR optimizes the balance between total coverage and repeat fidelity; the parameter L denotes the HOR length, defined as the total number of constituent monomeric units. HOR structure was successfully resolved for most centromeres across all eight species. Exceptions were *L. catta* (LCA) and *E. collaris* (ECO), for which HiCAT was applied to a single representative centromere per species due to the disproportionate size of their centromere arrays relative to their short monomer units (41 bp and 6 bp, respectively), which precluded full-genome HOR decomposition.

HORs were also identified using CENdetectHOR (Daponte et al., 2025). CENdetectHOR defines the resulting HORs using the nomenclature format, CnHn, where Cn represents the contig identification number (substituting the chromosome identifier typically used in anchored assemblies) and Hn indicates the length of the identified HOR unit. The analysis was conducted only on the two species, *Propithecus coquereli* and *Cheroilogaleus medius*, utilizing scaffold-level assemblies. A consensus sequence for the respective monomeric repeats was used as a reference. The analysis followed the standard detection protocol, using default parameters.

bioRxiv preprint doi: https://doi.org/10.64898/2026.05.16.725662; this version posted May 19, 2026. The copyright holder for this preprint (which was not certified by peer review) is the author/funder, who has granted bioRxiv a license to display the preprint in perpetuity. It is made available under a CC-BY 4.0 International license.

Visual and structural analysis of the outputs was performed using the PhyloTreeGUI graphical interface, which allowed for the reconstruction and inspection of HOR-based phylogenetic trees. The downstream analysis involved the systematic pruning of HOR trees to extract biologically significant structures. To ensure a balanced comparison, branches were selected at a consistent hierarchical level across the different trees. The selection process was guided by a coverage threshold, maintaining approximately 95% coverage of the repetitive regions.

For each assembled centromere, array size and sequence identity relative to the species-specific monomer were calculated. As a reference for comparative context with haplorhine centromeres, the canonical alpha satellite monomer sequence was used:

```
AATCTGCAAGTGGATATTTGGACCGCTTTGAGGCCTTCGTTGGAAACGGGAATATCTTCATATAAAAACTAGA
CAGAAGCATTCTCAGAAACTTCTTTGTGATGTGTGCATTCAACTCACAGAGTTGAACCTTCCTTTTCATAGAG
CAGTTTTGAAACACTCTTTTTGTAG
```

As all assemblies were fully phased or pseudo haplotype-resolved, allelic centromere pairs could be identified for each chromosome. Pairwise sequence identity was calculated between allelic centromeres (intra-individual) and between non-allelic centromeres (inter-individual), with unique single-copy genomic sequences used as a specificity control. Centromere sequences were aligned using minimap2 with the following command, according to Logsdon et al. (2024):

```
minimap2 -I 15G -K 8G -t {threads} -ax asm20 --secondary=no --eqx -s 2500
{ref.fasta} {query.fasta}
```

Alignment identity statistics were extracted from the resulting PAF files using rustybam (https://github.com/vollgerlab/rustybam).

Centromere arrays were extracted and visualized as pairwise sequence identity heatmaps using StainedGlass (Vollger et al., 2022). Window size was selected for each species proportionally to the species-specific monomer length to ensure biologically meaningful resolution of repeat structure.

D. Molecular Evolution of Centromere Proteins

Orthologs of CENP-A, CENP-B, and CENP-C were identified in each lemur assembly using miniprot, with *Lemur catta* reference protein sequences as queries (GCF_020740605.2). Coding sequences were extracted from the resulting alignments using gffread (-x flag) to obtain coding sequence (CDS) in the correct reading frame. Sequences were inspected for premature stop codons and frameshifts prior to alignment. We also procured CENP CDS for 15 other genome assembled species in NCBI: *Carlito syrichta* (GCF_000164805.1), *Rhinopithecus bieti* (GCF_001698545.2), *Papio anubis* (GCF_008728515.1), *Trachypithecus francoisi* (GCF_009764315.1), *Pongo abelii* (GCF_028885655.2), *Pan paniscus* (GCF_029289425.2), *Macaca*

bioRxiv preprint doi: https://doi.org/10.64898/2026.05.16.725662; this version posted May 19, 2026. The copyright holder for this preprint (which was not certified by peer review) is the author/funder, who has granted bioRxiv a license to display the preprint in perpetuity. It is made available under a CC-BY 4.0 International license.

fascicularis (GCF_037993035.2), *Macaca mulatta* (GCF_049350105.2), *Chlorocebus sabaeus* (GCF_047675955.1), *Cebus imitator* (GCF_001604975.1), *Sapajus apella* (GCF_009761245.1), *Aotus nancymaae* (GCF_030222135.1), *Saimiri boliviensis* (GCF_048565385.1), *Callithix jacchus* (GCF_049354715.1), and *Homo sapiens* (GCF_009914755.1).

CDS were translated using *seqkit translate* (Shen et al., 2024), selecting the longest isoform in case of multiple isoforms with AGAT's agat_sp_keep_logest_isoform.pl script (Dainat et al., 2021), aligning the protein sequences using MAFFT (Katoh et al., 2019), and the resulting amino acid alignment was used to guide codon-aware back-translation of the corresponding nucleotide sequences using pal2nal (Suyama et al., 2006). The final codon alignments were used as input for all downstream selection analyses. All selection analyses were conducted on the species tree derived from Timetree5 (Kumar et al., 2022).

PAML codeml: Selection analyses were performed using codeml from the PAML package (Yang, 2007). A model M0 (one-ratio) analysis was first run for each gene to estimate the genome-wide background dN/dS (ω) ratio across all branches. Branch-site model analyses were then conducted to test for episodic positive selection along foreground (lemur) branches. The branch-site test compares a model allowing $\omega > 1$ on foreground branches (Model A) against a null model with ω constrained to 1, using a likelihood ratio test (LRT). Internal node labels were specified using the ete3 tree-manipulation framework to ensure correct foreground branch designation in the PAML control file. P-values were corrected for multiple testing using the Benjamini-Hochberg procedure.

RELAX: Relaxation or intensification of selection pressure on lemur lineages was assessed using RELAX (Wertheim et al., 2015) as implemented on the Datamonkey web server. RELAX fits a model with a selection intensity parameter K to foreground branches relative to reference branches, where $K > 1$ indicates intensified selection and $K < 1$ indicates relaxation. The root node was explicitly labelled as the reference branch (`{Reference}`) as required by RELAX. Statistical significance was assessed by LRT against a null model with K constrained to 1.

BUSTED: Evidence for episodic diversifying selection anywhere in the lemur foreground branches was tested using BUSTED (Murrell et al., 2015) as implemented on the Datamonkey web server. BUSTED tests whether at least one branch and site combination in the foreground has experienced positive selection ($\omega > 1$), without requiring that selection be pervasive. Results were considered significant at $p < 0.05$.

bioRxiv preprint doi: https://doi.org/10.64898/2026.05.16.725662; this version posted May 19, 2026. The copyright holder for this preprint (which was not certified by peer review) is the author/funder, who has granted bioRxiv a license to display the preprint in perpetuity. It is made available under a CC-BY 4.0 International license.

Supplementary Tables

Supplementary Table 1 – Assemblies of Lemuriformes in NCBI

Species	Assembly ID	# of contigs	Contig N50	Assembly level	Genome coverage	Reference
Lemur catta	GCF_020740605.2	394	32.5 Mbp	Chromosome	20.81x	Palmada-Flores et al., 2022
Varecia variegata	GCA_028533085.1	1,029,863	40.2 kbp	Chromosome	74x	DNA Zoo (unpublished)
Microcebus murinus	GCF_000165445.2	50,982	210.7 kbp	Chromosome	221.6x	Baylor College (Unpublished)
Varecia rubra	GCA_963573675.1	112,503	37.4 kbp	Scaffold	35x	Kuderna et al., 2023
Eulemur collaris (referenced as Eulemur fulvus collaris)	GCA_963575015.1	111,525	39 kbp	Scaffold	35x	Kuderna et al., 2023
Propithecus coquereli	GCF_000956105.1	299,069	28.1 kbp	Scaffold	104.7x	Baylor College (Unpublished)
Cheirogaleus medius	GCA_008086735.1	130,903	34.9 kbp	Scaffold	110x	Williams et al., 2020
Daubentonia madagascariensis	GCA_044048945.1	930	80.4 Mbp	Scaffold	135x	Versoza and Pfeifer, 2024

bioRxiv preprint doi: https://doi.org/10.64898/2026.05.16.725662; this version posted May 19, 2026. The copyright holder for this preprint (which was not certified by peer review) is the author/funder, who has granted bioRxiv a license to display the preprint in perpetuity. It is made available under a CC-BY 4.0 International license.

Supplementary Table 2 – NucFreq and Flagger statistics of the new assemblies

	Microcebus murinus	*Cheirogaleus medius*	*Propithecus coquereli*	*Daubentonia madagascariensis*	*Lemur catta*	*Varecia rubra*	*Varecia variegata*	*Eulemur collaris*
**NucFreq**								
Misassembly	3.08 Mbp	0.75 Mbp	1.33 Mbp	0.65 Mbp	0.37 Mbp	0.35 Mbp	0.18 Mbp	0.78 Mbp
Collapse	4.16 Mbp	0.92 Mbp	23.2 Mbp	0.66 Mbp	0.78 Mbp	0.20 Mbp	0.14 Mbp	0.19 Mbp
**Flagger_0.3.3**								
Erroneous	13.2 Mbp	2.86 Mbp	17.3 Mbp	7.09 Mbp	7.76 Mbp	1.57 Mbp	1.25 Mbp	8.25 Mbp
Duplicated	140 Mbp	93.1 Mbp	18.7 Mbp	43.1 Mbp	48.5 Mbp	34 Mbp	53.5 Mbp	141 Mbp
Collapses	9.92 Mbp	91.4 Mbp	26.3 Mbp	1.30 Mbp	15.8 Mbp	0.77 Mbp	0.64 Mbp	137 Mbp
Unknown	1.22 Mbp	0.32 Mbp	0.21 Mbp	0.94 Mbp	0.25 Mbp	0.09 Mbp	0.12 Mbp	0.30 Mbp
Haploid	4.57 Gbp	4.37 Gbp	4.60 Gbp	4.89 Gbp	4.49 Gbp	4.34 Gbp	4.35 Gbp	4.19 Gbp

bioRxiv preprint doi: https://doi.org/10.64898/2026.05.16.725662; this version posted May 19, 2026. The copyright holder for this preprint (which was not certified by peer review) is the author/funder, who has granted bioRxiv a license to display the preprint in perpetuity. It is made available under a CC-BY 4.0 International license.

Supplementary Table 3 - Mapping between chromosome numbers of *Lemur catta* karyotype from Cardone et al., 2002 and NCBI assembly

Cardone et al., 2002	Lemur_NCBI
1	NC_059128.1
2	NC_059129.1
3	NC_059130.1
4	NC_059132.1
5	NC_059131.1
6	NC_059139.1
7	NC_059135.1
8	NC_059134.1
9	NC_059138.1
10	NC_059133.1
11	NC_059136.1
12	NC_059141.1
13	NC_059137.1
14	NC_059140.1
15	NC_059144.1
16	NC_059147.1
17	NC_059142.1
18	NC_059143.1
19	NC_059146.1
20	NC_059145.1
21	NC_059148.1
22*	NC_059150.1
23*	NC_059151.1
24*	NC_059152.1
25	NC_059149.1
26*	NC_059153.1
27*	NC_059154.1
X	NC_059155.1
Y	NC_059156.1

bioRxiv preprint doi: https://doi.org/10.64898/2026.05.16.725662; this version posted May 19, 2026. The copyright holder for this preprint (which was not certified by peer review) is the author/funder, who has granted bioRxiv a license to display the preprint in perpetuity. It is made available under a CC-BY 4.0 International license.

Supplementary Table 4 - Primers used for probe synthesis. Annealing temperatures and expected product sizes are provided for each species-specific monomer targeting putative centromeric regions

**Species**	**Target Sequence**	**Primer sequence (5' → 3')**	**Annealing T (°C)**	**Probe size (bp)**
Coquerel's sifaka (PCO)	Pc170	PCO_170mer_F 5’-CCTGCTTTCTTACGTGTGTTTCA-3’ PCO_170mer_R 5’-TCTCTCAGAGAAAGCAGCTAGAC-3’	56	106
	PcYcen sequence	PCO_Ycen_F 5’-GGGCAGCACCGCTTAGTTACAGAA-3’ PCO_Ycen_R 5’- CGCAGTGGGCTTCCGTACGA-3’	60	228
Ring-tailed lemur (LCA)	Lc41	LCA_41mer_F 5’-AAGAGAACCCCAACCCTTACCA-3’ LCA_41mer_R 5’-GGGTTCAGGTGTGCATTAAGCTTTA-3’	60	170
Black and white ruffed lemur (VVA)	Vv166	VVA_166mer_F 5’-TGCAATCCCACCACTGTTAGT-3’ VVA_166mer_R 5’-ACAGAAGTCATTTCGCAGGAAGA-3’	56	106
	Vv1399	VVA_1399mer_F 5’-GTTCTGGTTCTCGTTCGGGT-3’ VVA_1399mer_R 5’-TCCGGTGATGCTCCAAATCC-3’	56	603
Collared brown lemur (ECO)	Ec548	ECO_548mer_F 5’-GTCACTGGAGGGGACGTTTT-3’ ECO_548mer_R 5’-GCACTTTTCACCTGCCACAC-3’	55	128

bioRxiv preprint doi: https://doi.org/10.64898/2026.05.16.725662; this version posted May 19, 2026. The copyright holder for this preprint (which was not certified by peer review) is the author/funder, who has granted bioRxiv a license to display the preprint in perpetuity. It is made available under a CC-BY 4.0 International license.

Supplementary Table 5 - CENdetectHOR results for the T2T contigs of two species: CME and PCO

T2T Contig	HOR name	length	Abundancy (bp)	% Abundancy
**PCO_mat0000005**	C5H2	2	3,706,083	80.1%
	C5H17	17	139,257	3.0%
	C5H27	27	101,681	2.2%
**PCO_mat0000007**	C7H2.1	2	4,635,132	80.6%
	C7H24	24	88,928	1.5%
	C7H11	11	42,877	0.7%
**PCO_mat0000010**	C10H2	2	3,044,541	87.0%
	C10H30	30	56,847	1.6%
	C10H3	3	33,437	1.0%
**PCO_mat0000021**	C21H2	2	2,351,724	62.8%
	C21H5	5	212,677	5.7%
	C21H7	7	156,039	4.2%
**PCO_mat0000026**	C26H2	2	2,260,488	86.7%
	C26H27	27	39,269	1.5%
	C26H36	36	24,586	0.9%
**PCO_mat0000030**	C30H2	2	2,058,986	84.8%
	C30H20	20	124,384	5.1%
	C30H11	11	34,347	1.4%
**CME.h1tg000007l**	C7lH12	12	396,018	74.8%
	C7lH24	24	21,925	4.1%
	C7lH36	36	16,928	3.2%
**CME.h1tg000013l**	C13lF1	1	6,946	54.1%
	C13lH3	3	2,709	21.1%
	C13lH4	4	2,295	17.9%
**CME.h1tg000029l**	C29lF1	1	12,773	86.1%
	C29lH4	4	1,858	12.5%
**CME.h1tg000010l**	C10lH7	7	184,538	33.0%
	C10lH7.3	7	28,808	5.1%
	C10lH23	23	25,024	4.5%
**CME.h1tg000024l**	C24lH12	12	795,040	79.8%
	C24lH3	3	79,443	8.0%
	C24lH9	9	13,136	1.3%
**CME.h1tg00005l**	C5lH15	15	245,945	42.1%
	C5lH27	27	55,649	9.5%
	C5lH24	24	26,967	4.6%

bioRxiv preprint doi: https://doi.org/10.64898/2026.05.16.725662; this version posted May 19, 2026. The copyright holder for this preprint (which was not certified by peer review) is the author/funder, who has granted bioRxiv a license to display the preprint in perpetuity. It is made available under a CC-BY 4.0 International license.

Supplementary Figures

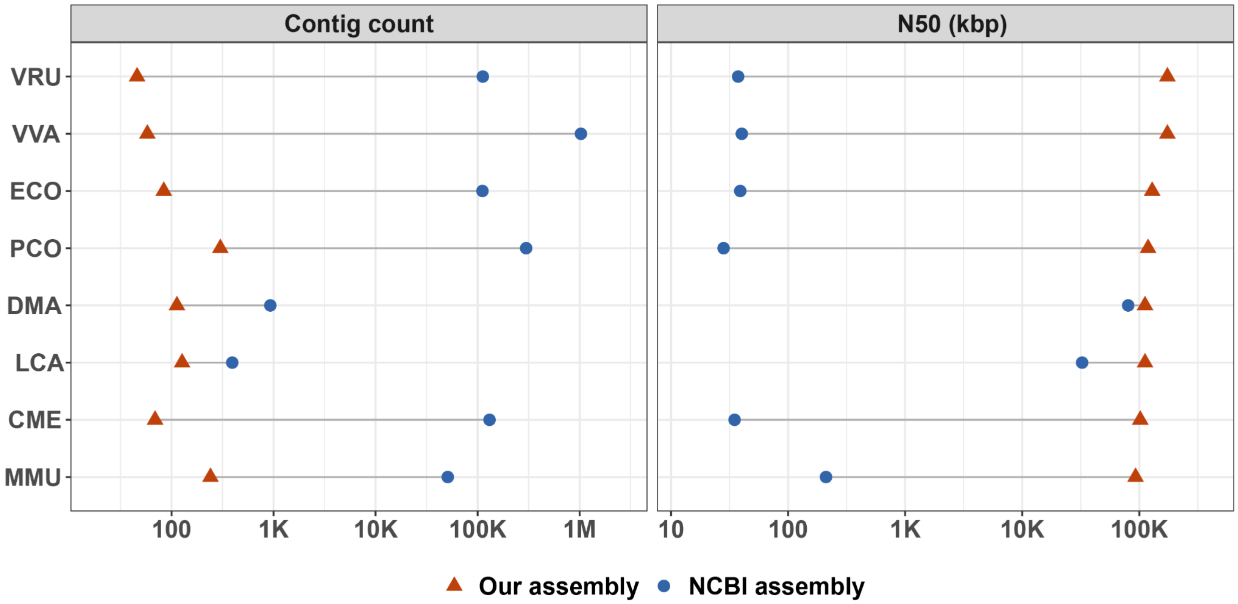

Figure S1. Improvement in assembly contiguity in this study. Comparison between the assemblies already submitted in the NCBI database for these lemur species, and the assemblies presented in this study. It can be clearly seen that both contig count and contig N50 sizes have drastically improved in our assemblies.

bioRxiv preprint doi: https://doi.org/10.64898/2026.05.16.725662; this version posted May 19, 2026. The copyright holder for this preprint (which was not certified by peer review) is the author/funder, who has granted bioRxiv a license to display the preprint in perpetuity. It is made available under a CC-BY 4.0 International license.

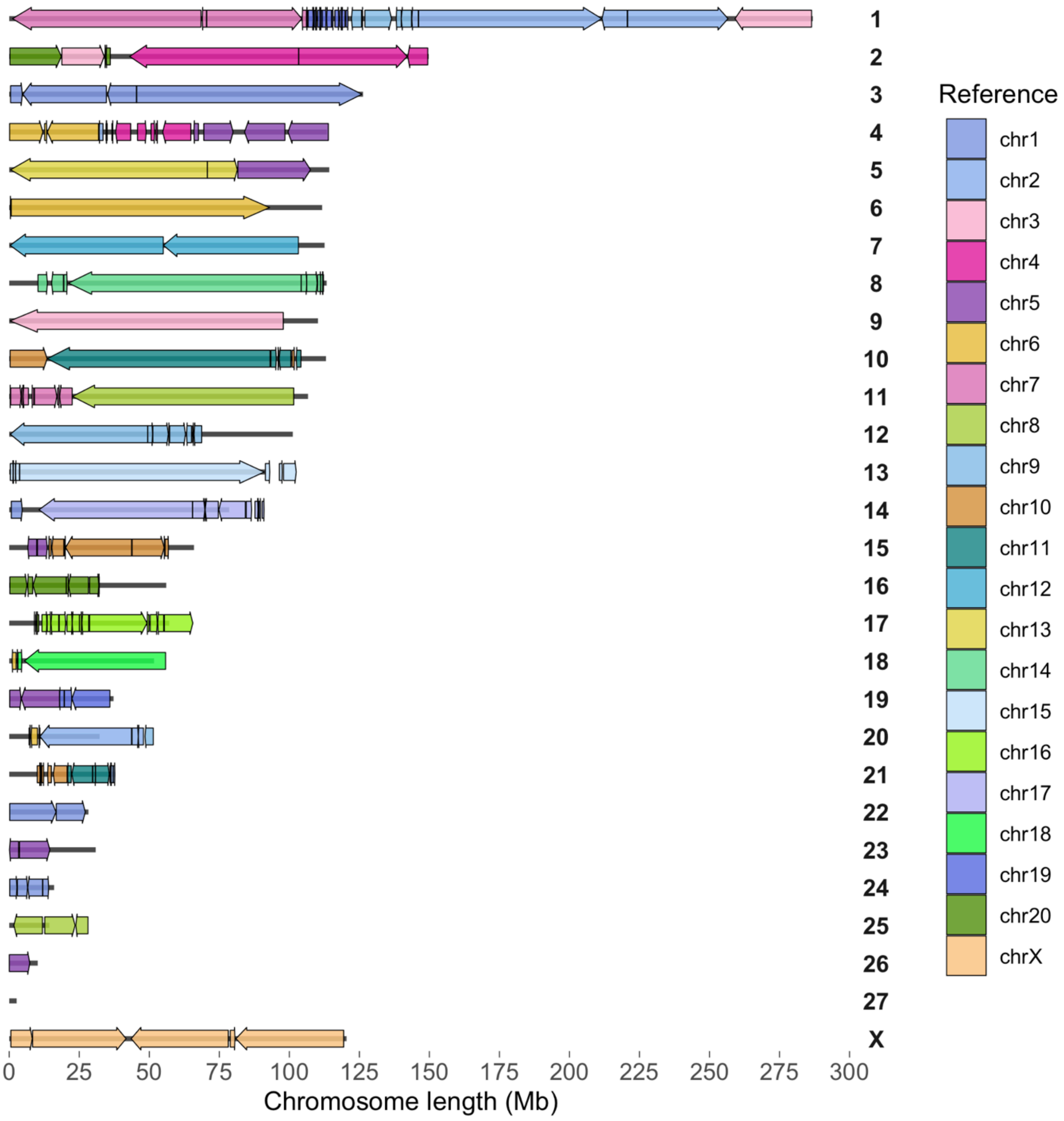

Figure S2. Synteny of *Lemur catta* genome with Macaca fascicularis (MFA). Synteny between MFA and *Lemur catta* (LCA) chromosomes, with LCA on the left and MFA chromosome colors on the right. Arrows show the orientation of MFA synteny blocks and straight lines showing the regions with no syntenic matches. There are six chromosomes which are fully conserved in both the species, viz., 7 (chr14 in MFA), 8 (chr12), 10 (chr15), 15 (chr16), 20 (chr20) and X. Even within these chromosomes rearrangements are visible in both p and q arms with centromere position.

bioRxiv preprint doi: https://doi.org/10.64898/2026.05.16.725662; this version posted May 19, 2026. The copyright holder for this preprint (which was not certified by peer review) is the author/funder, who has granted bioRxiv a license to display the preprint in perpetuity. It is made available under a CC-BY 4.0 International license.

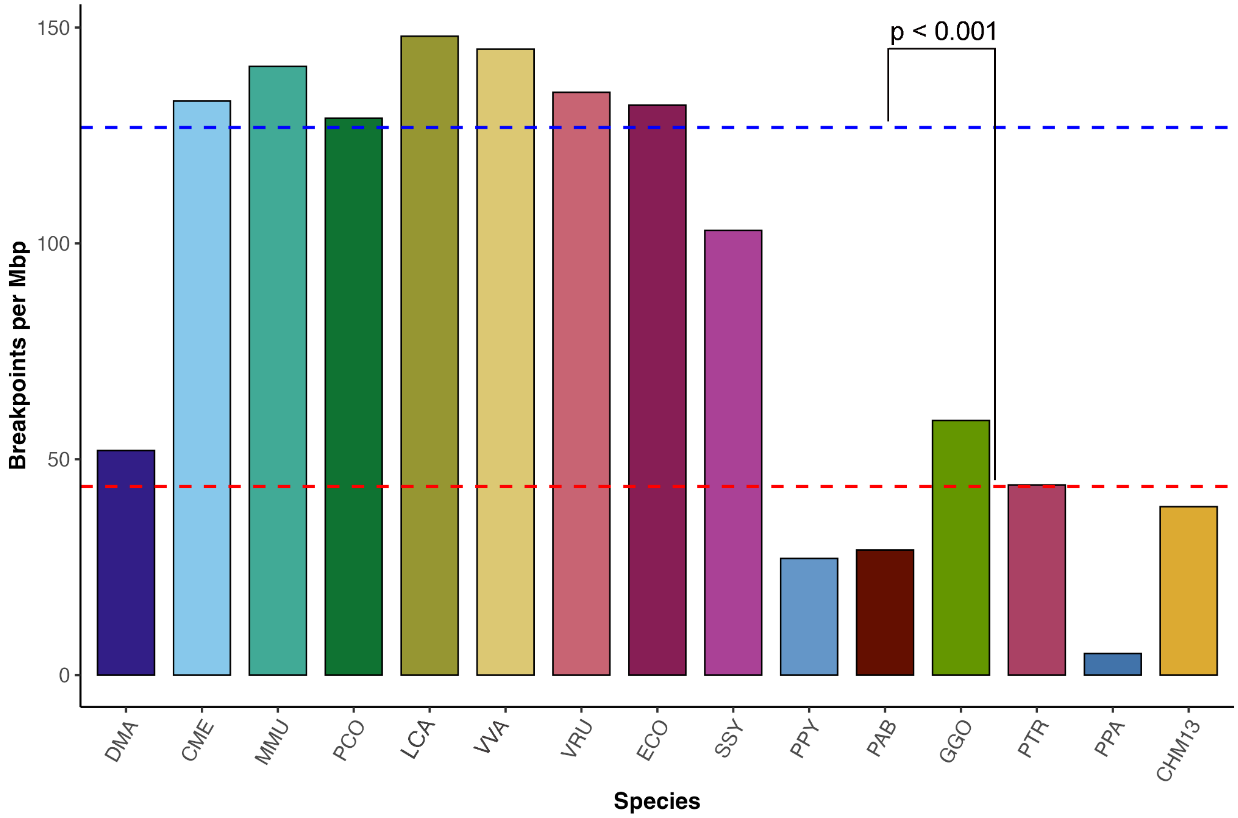

Figure S3. Breakpoints of lemur genome with respect to MFA. The plot displays the total breakpoint count for each species in this study and ape T2T genomes, using MFA as the reference. Consistent with T2T-CHM13-based comparisons, lemurs exhibit significantly higher breakpoint frequencies than haplorrhines when aligned to MFA ($p < 0.001$). The aye-aye (DMA) represents an outlier with markedly fewer breakpoints, likely due to limited alignment coverage (~200 Mbp aligned sequence).

bioRxiv preprint doi: https://doi.org/10.64898/2026.05.16.725662; this version posted May 19, 2026. The copyright holder for this preprint (which was not certified by peer review) is the author/funder, who has granted bioRxiv a license to display the preprint in perpetuity. It is made available under a CC-BY 4.0 International license.

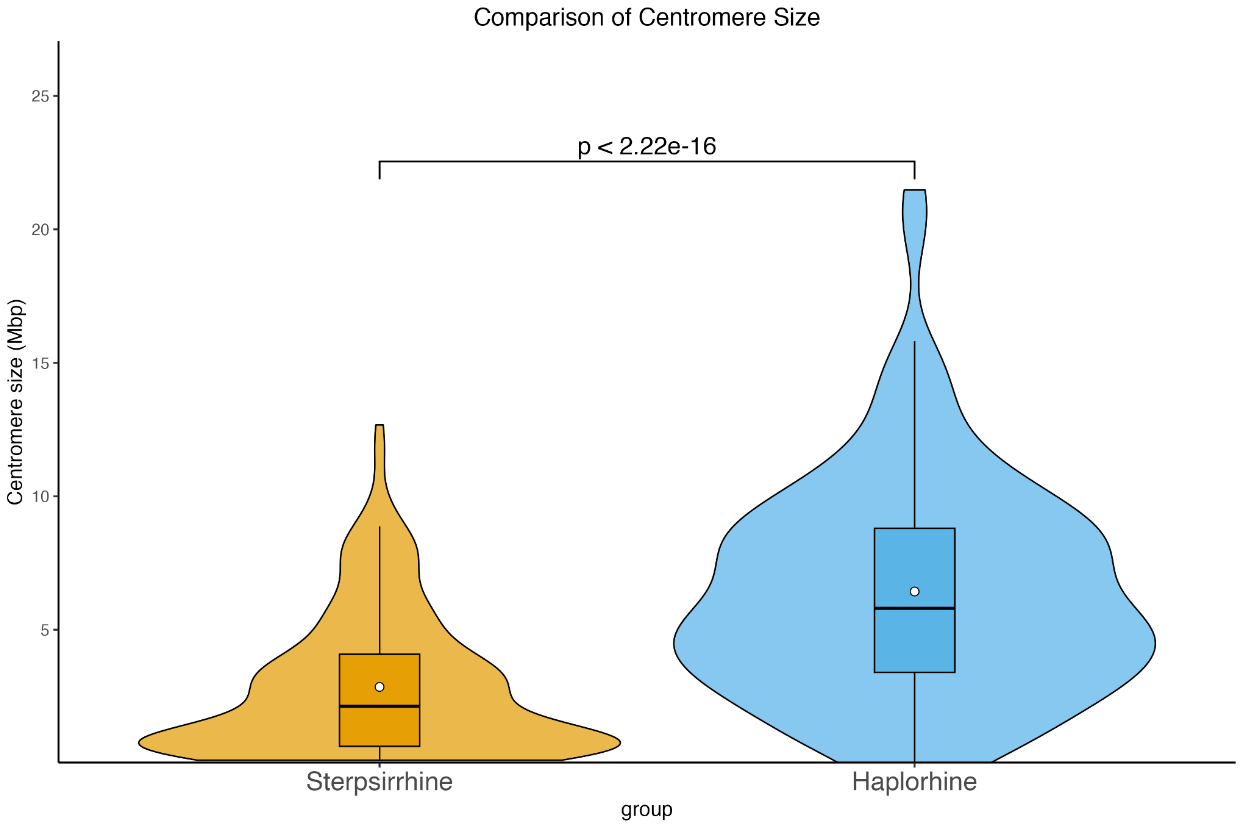

Figure S4. Centromere size comparison in strepsirrhines and haplorhines. We grouped the centromere sizes in two groups: strepsirrhine (eight lemur species) and haplorhine (MFA, apes and T2T-CHM13) and plotted their distributions. The difference is highly significant showing that strepsirrhine centromeres are smaller than haplorhines.

bioRxiv preprint doi: https://doi.org/10.64898/2026.05.16.725662; this version posted May 19, 2026. The copyright holder for this preprint (which was not certified by peer review) is the author/funder, who has granted bioRxiv a license to display the preprint in perpetuity. It is made available under a CC-BY 4.0 International license.

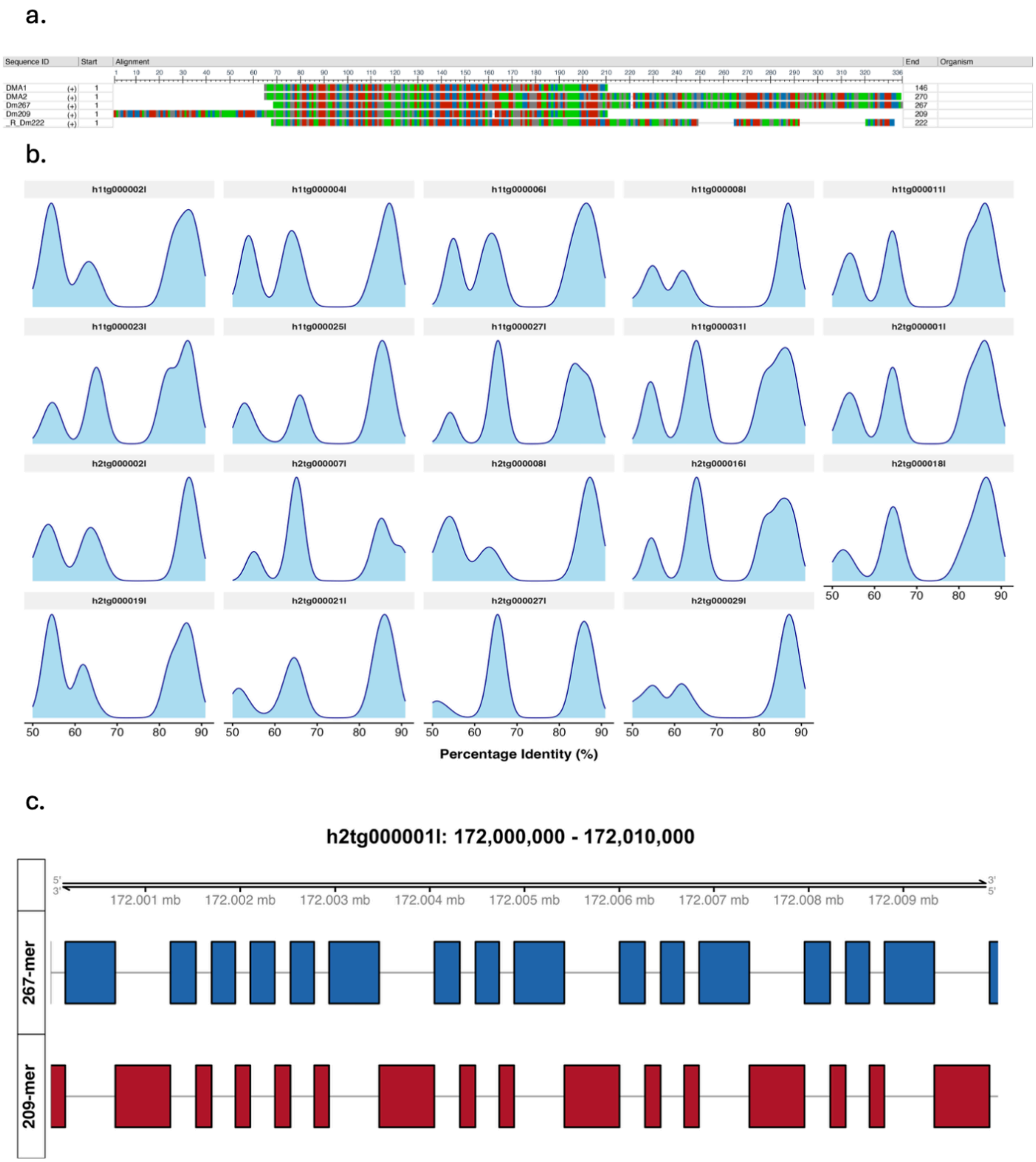

Figure S5. Detailed features of *Daubentonia madagascariensis* centromere. Features of DMA centromeres. a, Alignment of the three monomers found in this study along with the two monomers found in Lee et al., 2011. b, Density plots of identity of each complete centromere in the genome with respect to the most frequent canonical monomer, Dm267. c, High-resolution structure of one of the centromeres showing the repeats, Dm267 and Dm209, interspersed with each other.

bioRxiv preprint doi: https://doi.org/10.64898/2026.05.16.725662; this version posted May 19, 2026. The copyright holder for this preprint (which was not certified by peer review) is the author/funder, who has granted bioRxiv a license to display the preprint in perpetuity. It is made available under a CC-BY 4.0 International license.

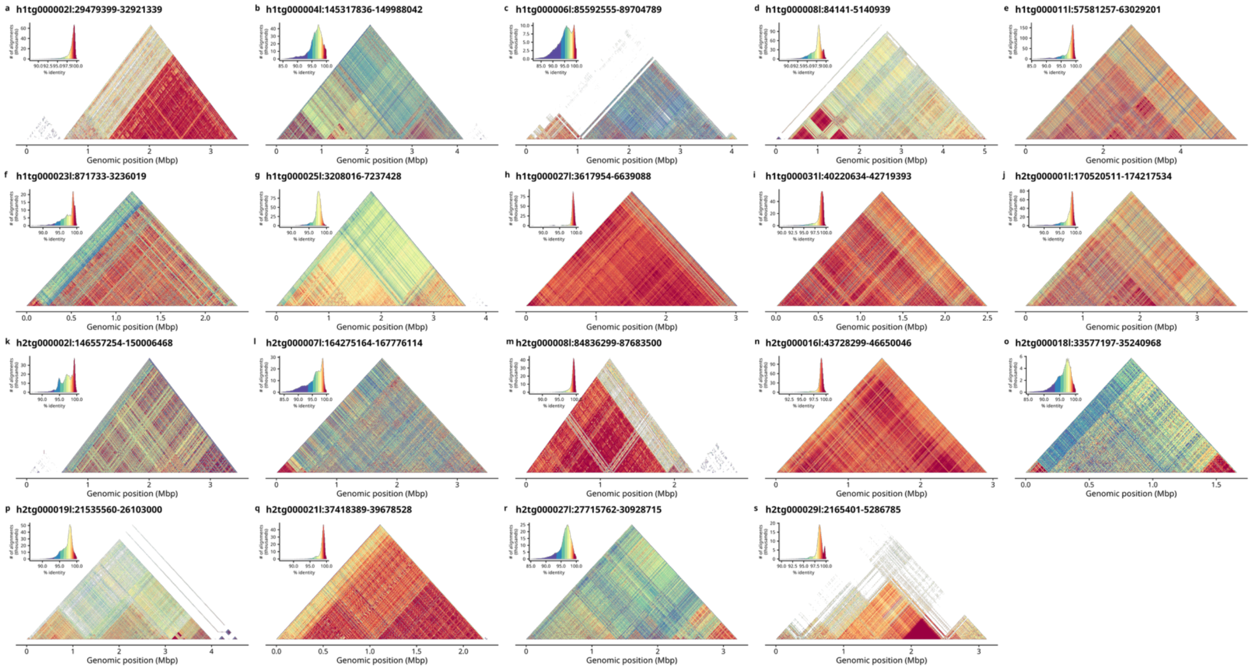

Figure S6. StainedGlass heatmaps for all assembled centromeres of *Daubentonia madagascariensis*

bioRxiv preprint doi: https://doi.org/10.64898/2026.05.16.725662; this version posted May 19, 2026. The copyright holder for this preprint (which was not certified by peer review) is the author/funder, who has granted bioRxiv a license to display the preprint in perpetuity. It is made available under a CC-BY 4.0 International license.

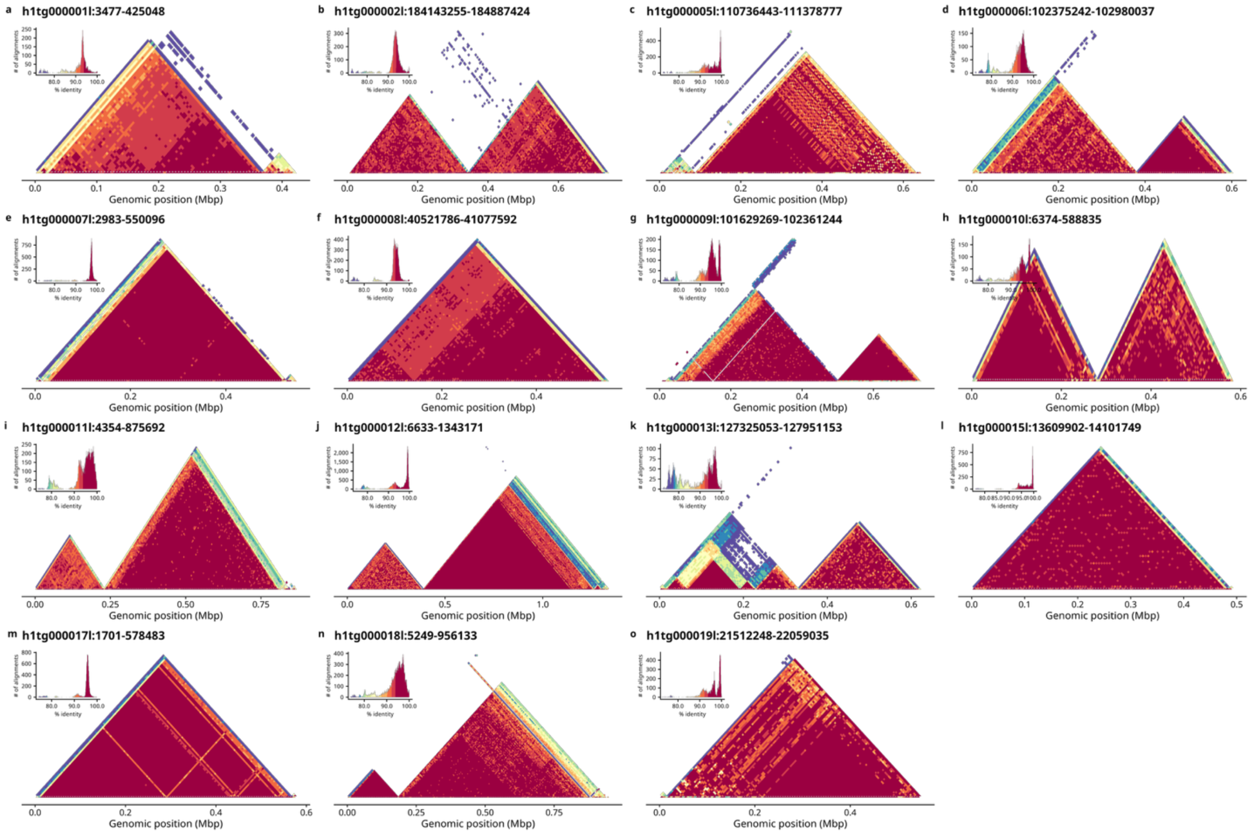

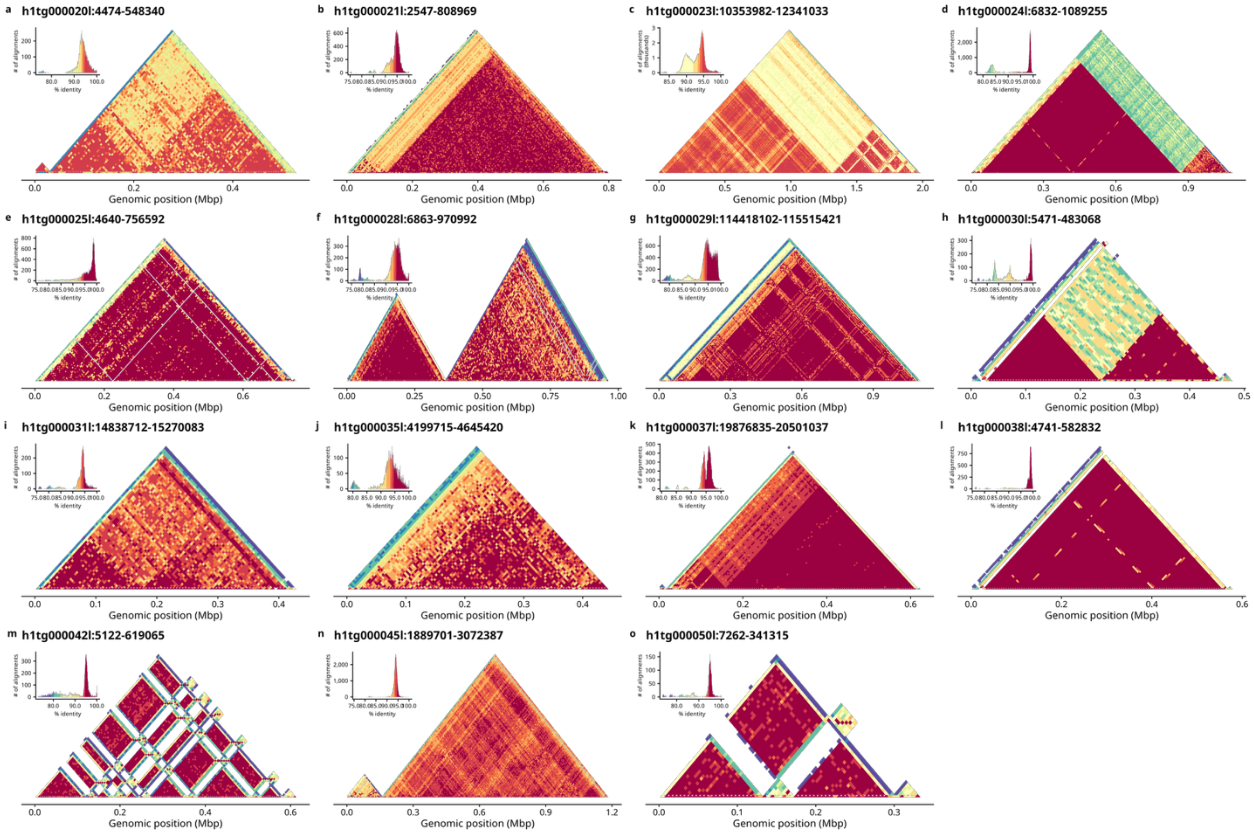

bioRxiv preprint doi: https://doi.org/10.64898/2026.05.16.725662; this version posted May 19, 2026. The copyright holder for this preprint (which was not certified by peer review) is the author/funder, who has granted bioRxiv a license to display the preprint in perpetuity. It is made available under a CC-BY 4.0 International license.

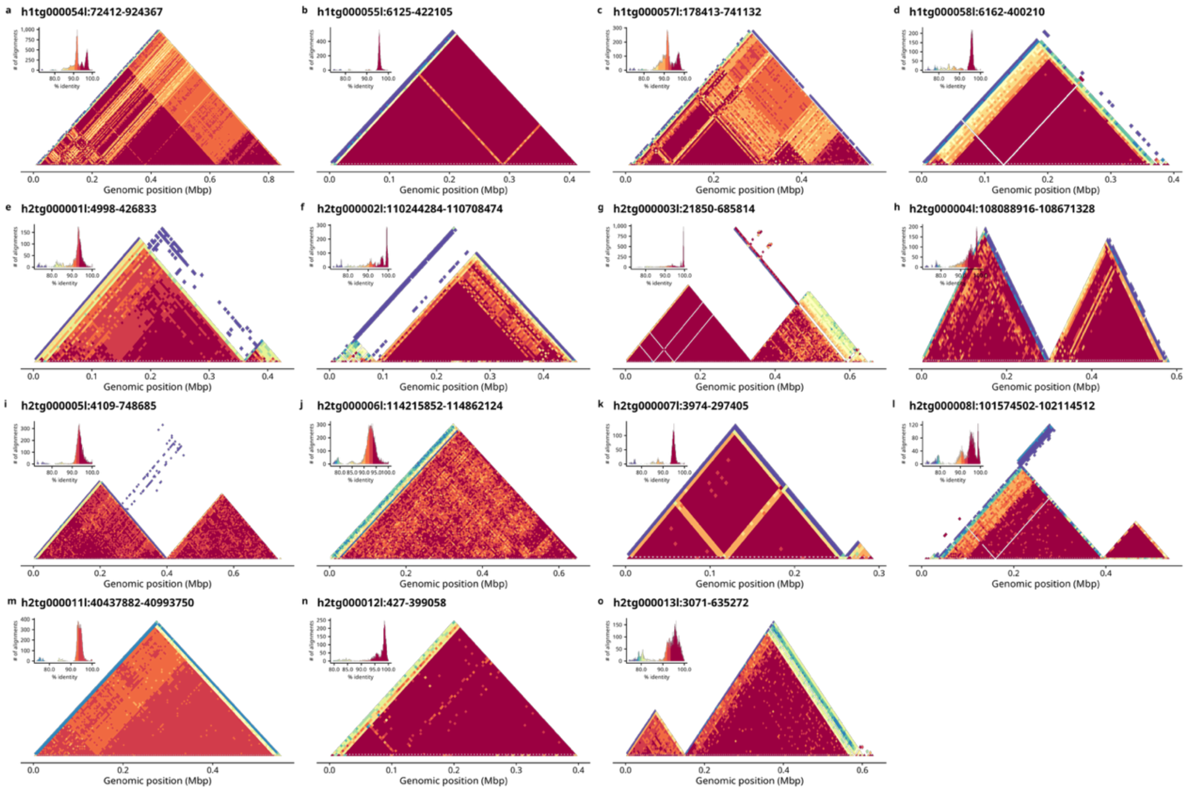

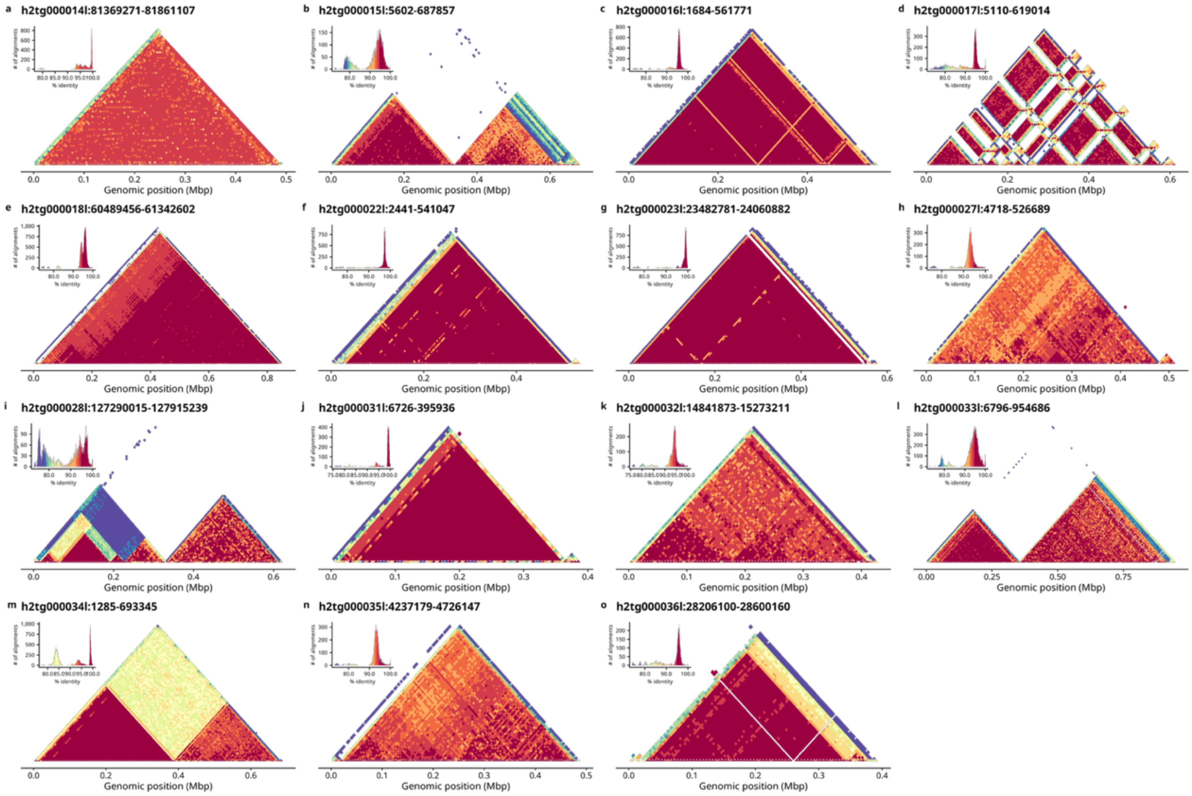

bioRxiv preprint doi: https://doi.org/10.64898/2026.05.16.725662; this version posted May 19, 2026. The copyright holder for this preprint (which was not certified by peer review) is the author/funder, who has granted bioRxiv a license to display the preprint in perpetuity. It is made available under a CC-BY 4.0 International license.

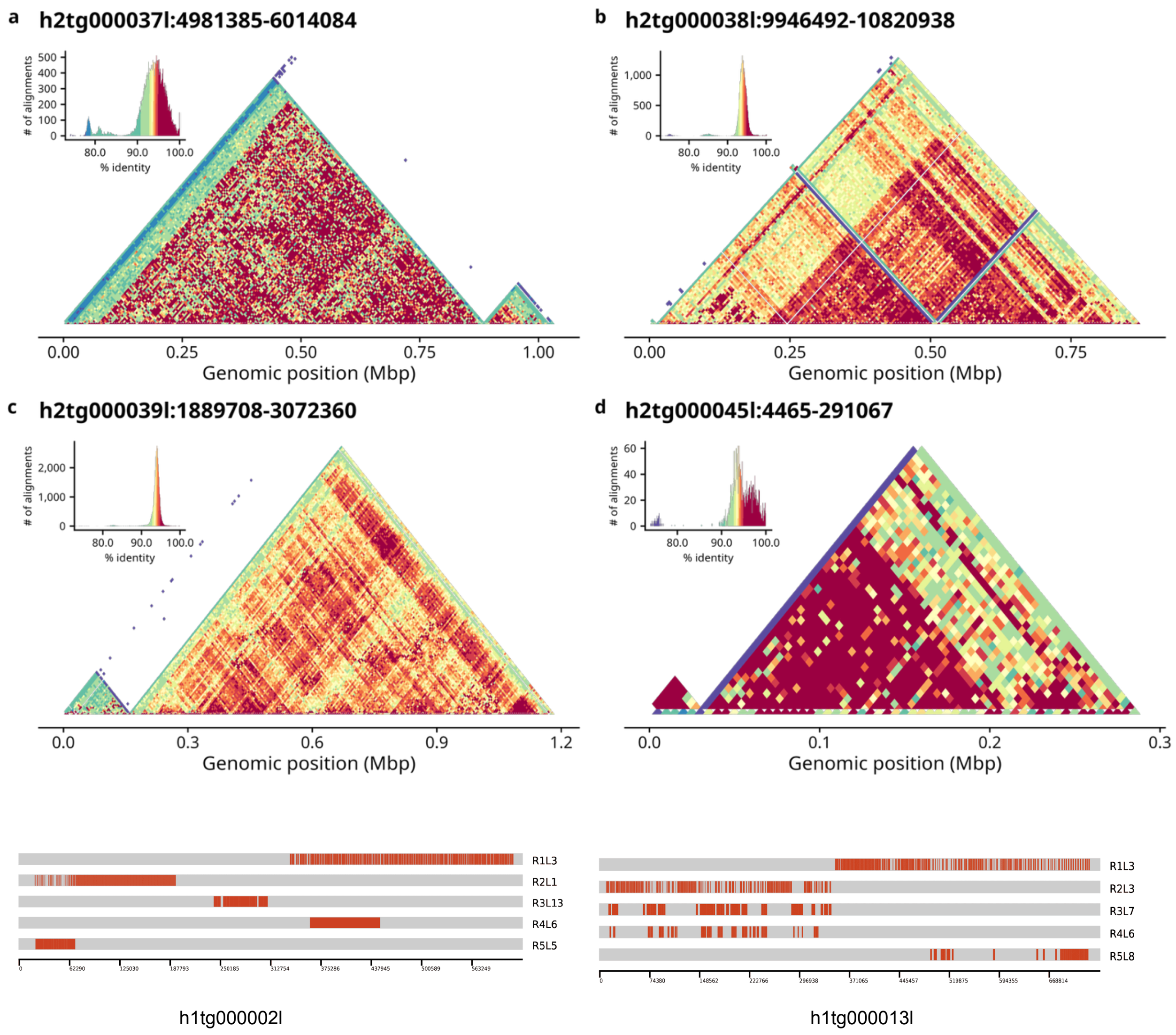

Figure S7. StainedGlass heatmaps for all the centromeres of *Cheirogaleus medius.* Heatmaps for centromeres of CME. The double "triangle" structure is made of two different HOR structures of the same Cm143 monomer. Examples of HOR structures for two centromeres are shown below the heatmap panel.

bioRxiv preprint doi: https://doi.org/10.64898/2026.05.16.725662; this version posted May 19, 2026. The copyright holder for this preprint (which was not certified by peer review) is the author/funder, who has granted bioRxiv a license to display the preprint in perpetuity. It is made available under a CC-BY 4.0 International license.

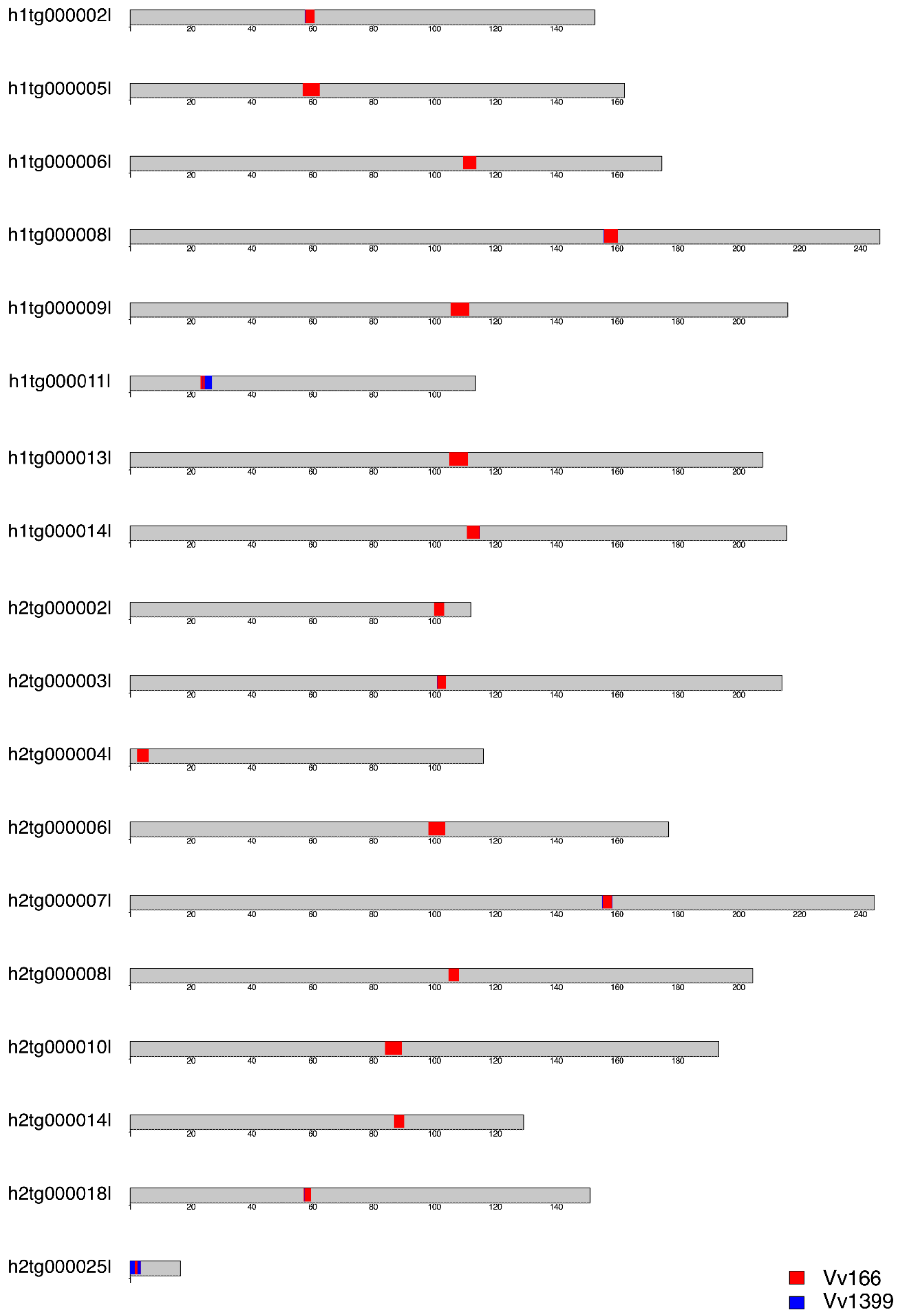

Figure S8. Ideogram showing centromeres on contigs of *Varecia variegata* (VVA). Ideogram showing the centromeres in each contig, with their repeat composition, whether they consist of Vv166 or Vv1405. Three types can be observed, made with either one of the two, or fused like h1tg000011l.

bioRxiv preprint doi: https://doi.org/10.64898/2026.05.16.725662; this version posted May 19, 2026. The copyright holder for this preprint (which was not certified by peer review) is the author/funder, who has granted bioRxiv a license to display the preprint in perpetuity. It is made available under a CC-BY 4.0 International license.

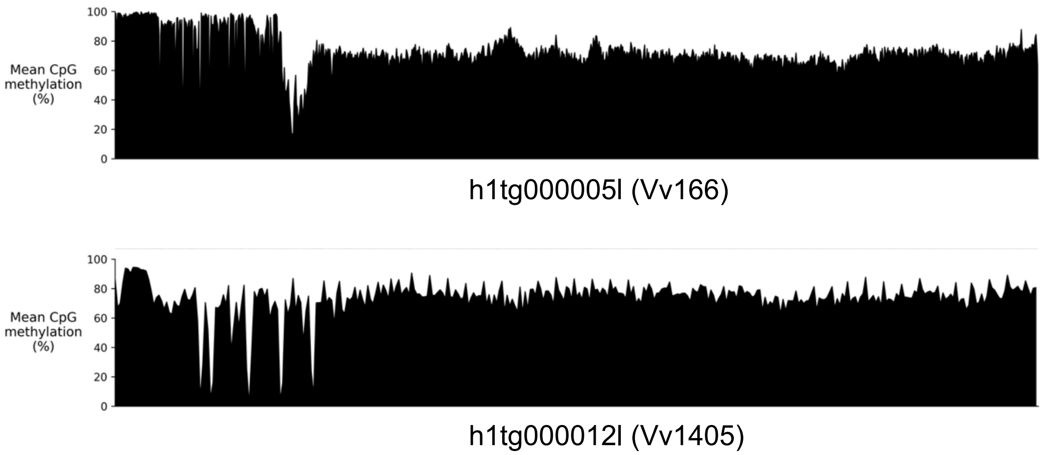

Figure S9. Methylation profiles of VVA centromeres consisting of different monomers. The difference in the methylation profiles of centromeres consists of Vv166 with a single CDR (above-h1tg000005l) and other with ragged CDR consisting of Vv1399 with putative small Vv166 arrays (below-h1tg000012l).

bioRxiv preprint doi: https://doi.org/10.64898/2026.05.16.725662; this version posted May 19, 2026. The copyright holder for this preprint (which was not certified by peer review) is the author/funder, who has granted bioRxiv a license to display the preprint in perpetuity. It is made available under a CC-BY 4.0 International license.

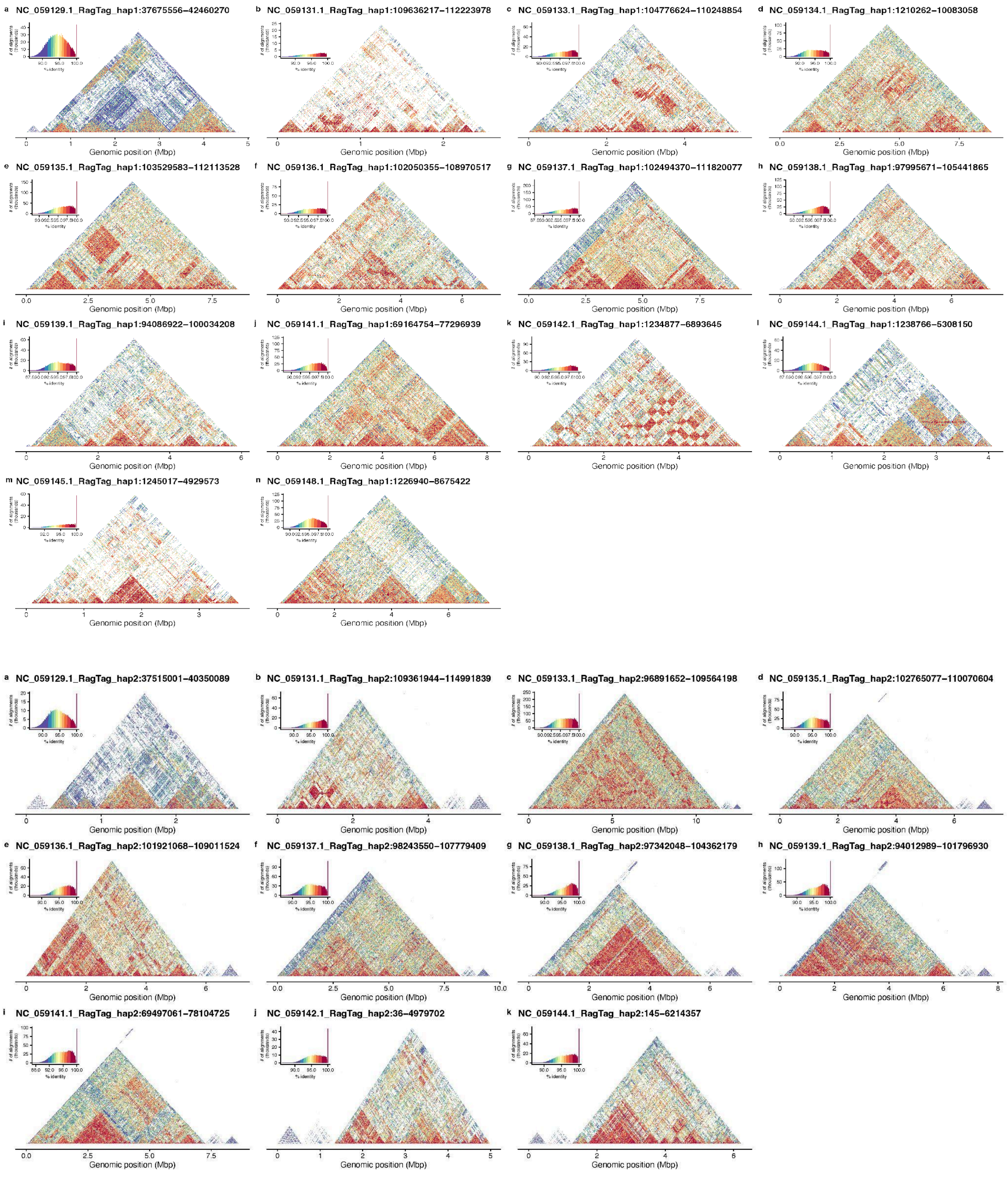

Figure S10. StainedGlass heatmaps for all the centromeres of *Lemur catta*. Heatmaps of all the complete centromeres of LCA. As described in the main text, the average identity of monomers of Lc41 is low due to the small size of the monomer and additionally could be because of the presence of partial polymers of 76 and 114 bp.

bioRxiv preprint doi: https://doi.org/10.64898/2026.05.16.725662; this version posted May 19, 2026. The copyright holder for this preprint (which was not certified by peer review) is the author/funder, who has granted bioRxiv a license to display the preprint in perpetuity. It is made available under a CC-BY 4.0 International license.

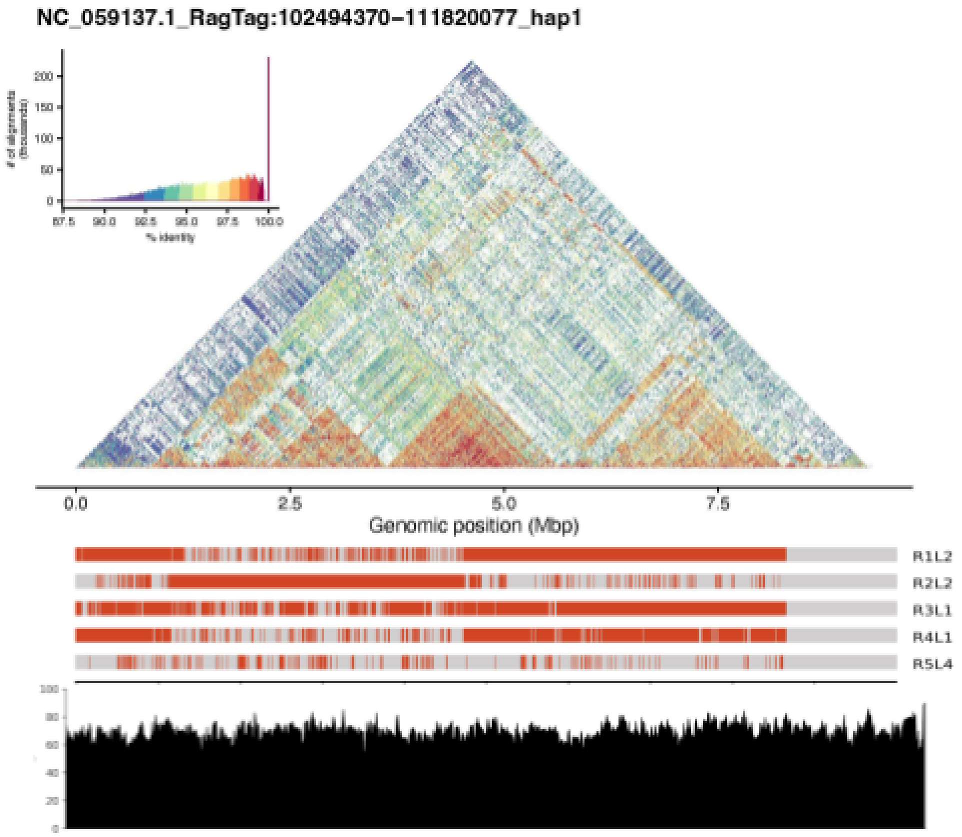

Figure S11. Structure of a single *Lemur catta centromere.* Here the centromere is showing an absence of a prominent CDR. Overall structure of an LCA centromere. The methylation profile does not show any prominent CDR and the whole profile is jagged.

bioRxiv preprint doi: https://doi.org/10.64898/2026.05.16.725662; this version posted May 19, 2026. The copyright holder for this preprint (which was not certified by peer review) is the author/funder, who has granted bioRxiv a license to display the preprint in perpetuity. It is made available under a CC-BY 4.0 International license.

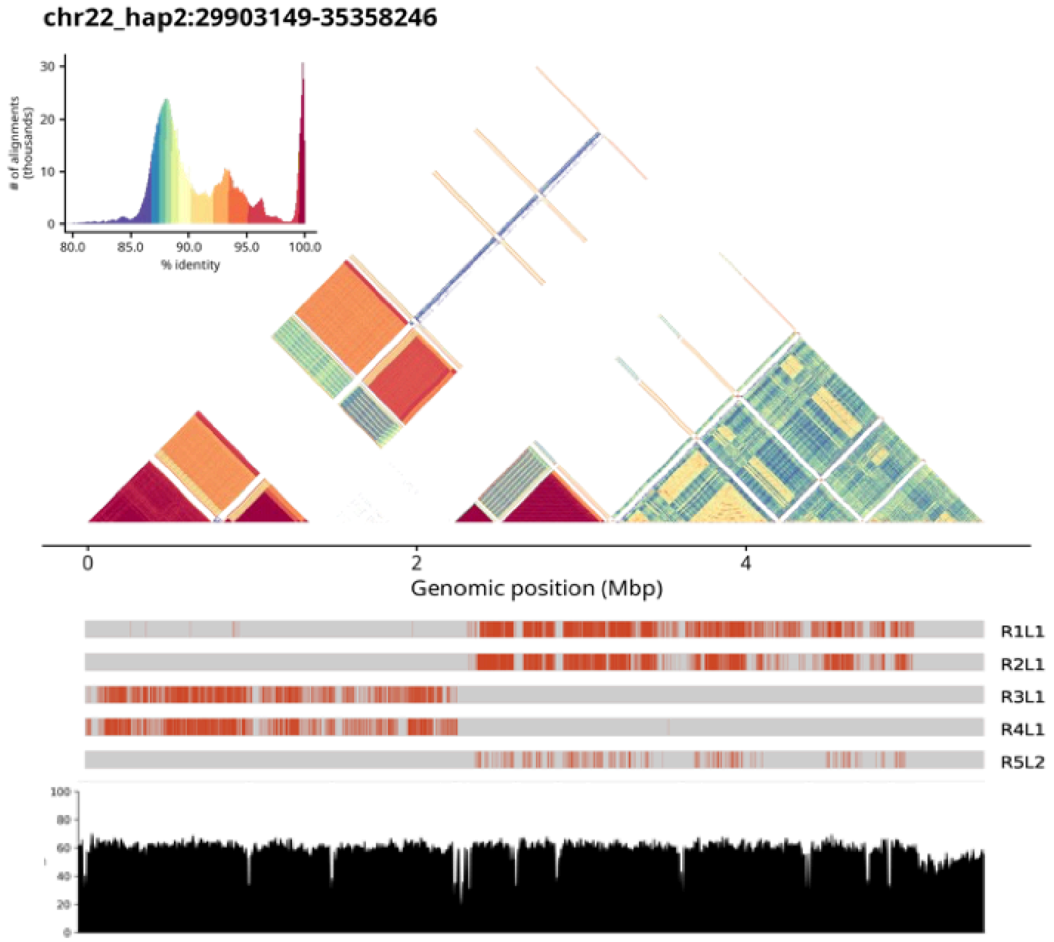

Figure S12. Structure of a single *Microcebus murinus* centromere. There are clearly two kinds of HORs in the centromere. They are not multi-monomeric and are only differentiated with their ranks. The methylation profile has multiple regions of hypomethylation dips.

bioRxiv preprint doi: https://doi.org/10.64898/2026.05.16.725662; this version posted May 19, 2026. The copyright holder for this preprint (which was not certified by peer review) is the author/funder, who has granted bioRxiv a license to display the preprint in perpetuity. It is made available under a CC-BY 4.0 International license.

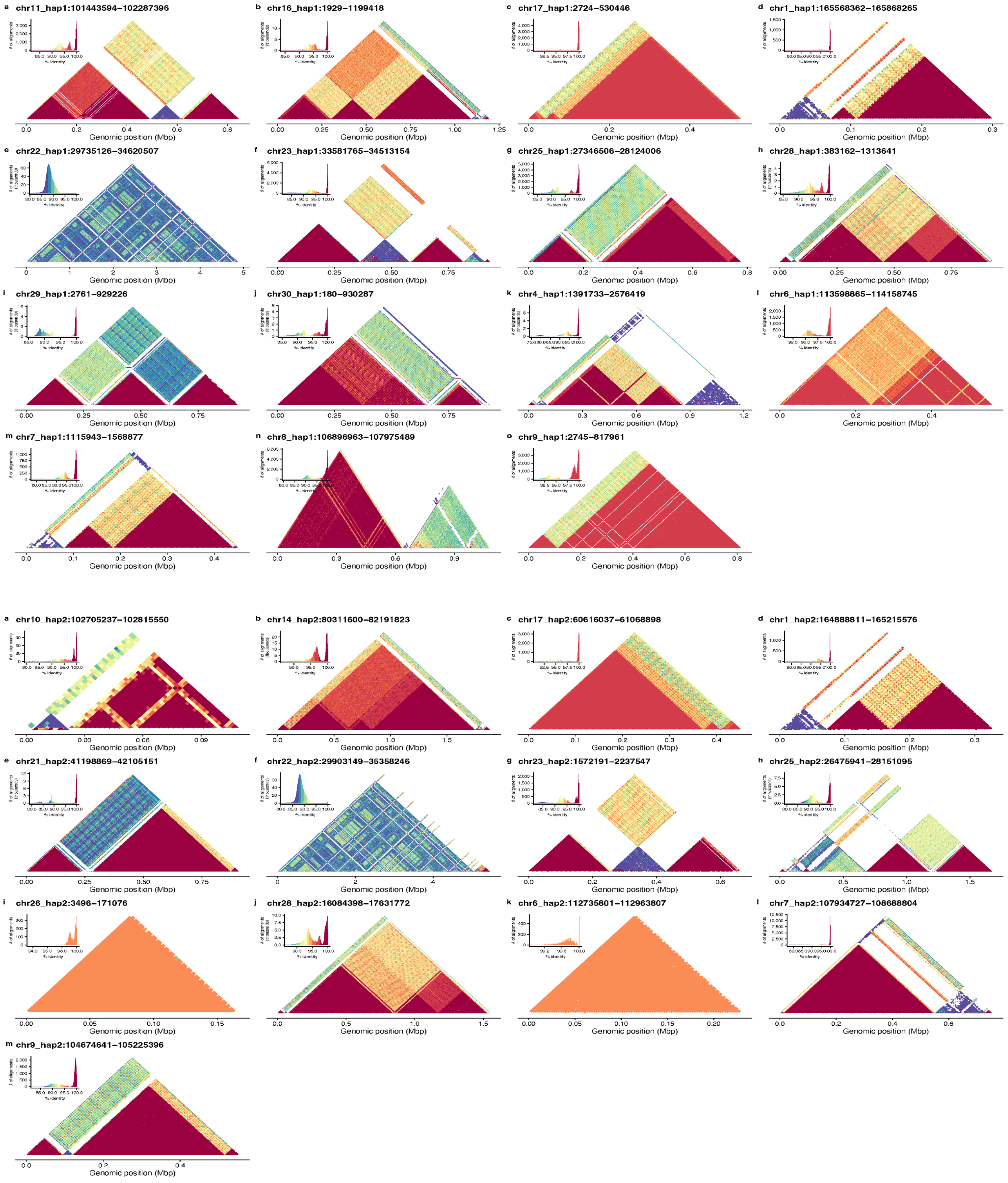

Figure S13 – StainedGlass heatmaps for all the centromeres of *Microcebus murinus*

bioRxiv preprint doi: https://doi.org/10.64898/2026.05.16.725662; this version posted May 19, 2026. The copyright holder for this preprint (which was not certified by peer review) is the author/funder, who has granted bioRxiv a license to display the preprint in perpetuity. It is made available under a CC-BY 4.0 International license.

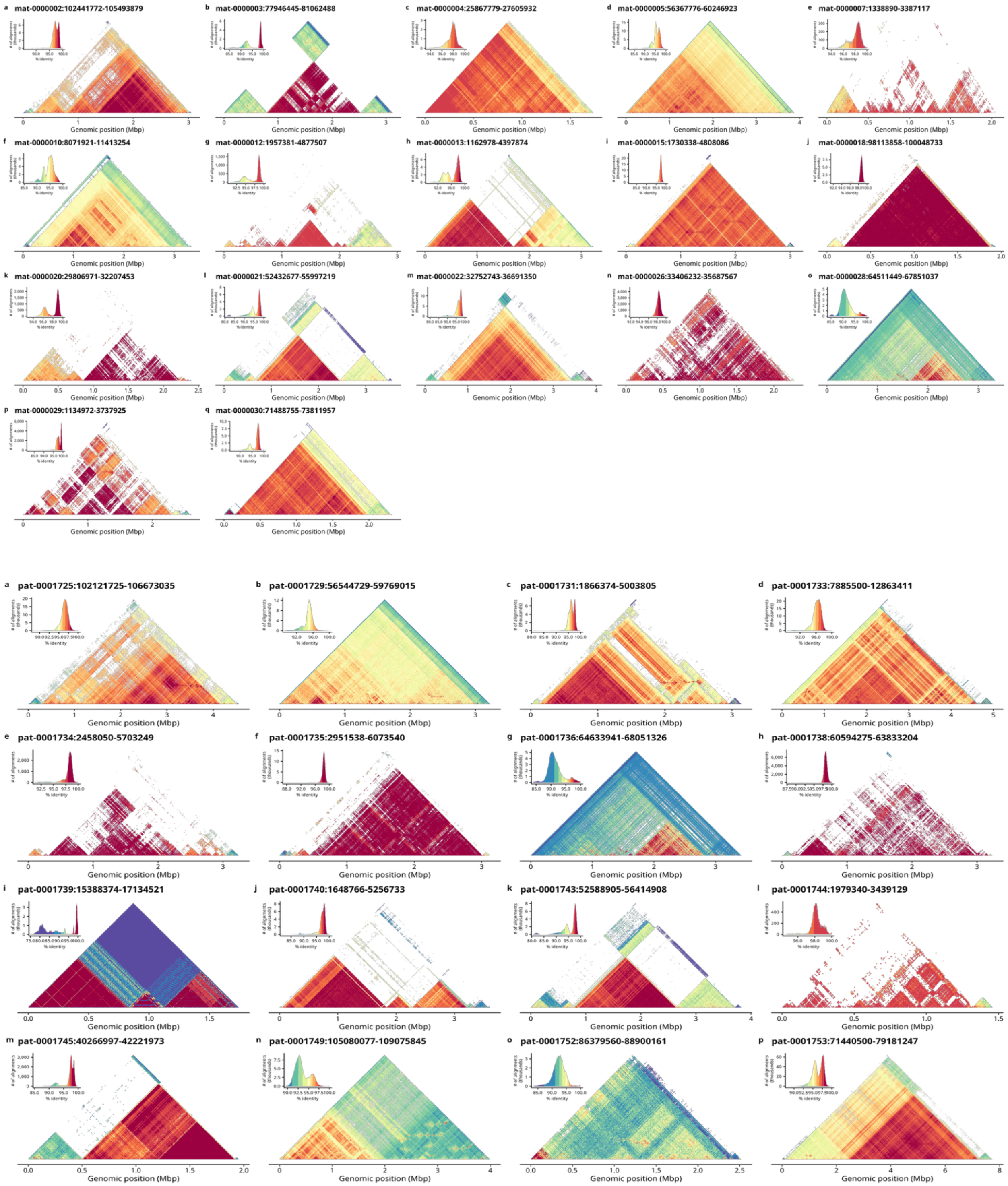

Figure S14. StainedGlass heatmaps for all the centromeres of *Propithecus coquereli*

bioRxiv preprint doi: https://doi.org/10.64898/2026.05.16.725662; this version posted May 19, 2026. The copyright holder for this preprint (which was not certified by peer review) is the author/funder, who has granted bioRxiv a license to display the preprint in perpetuity. It is made available under a CC-BY 4.0 International license.

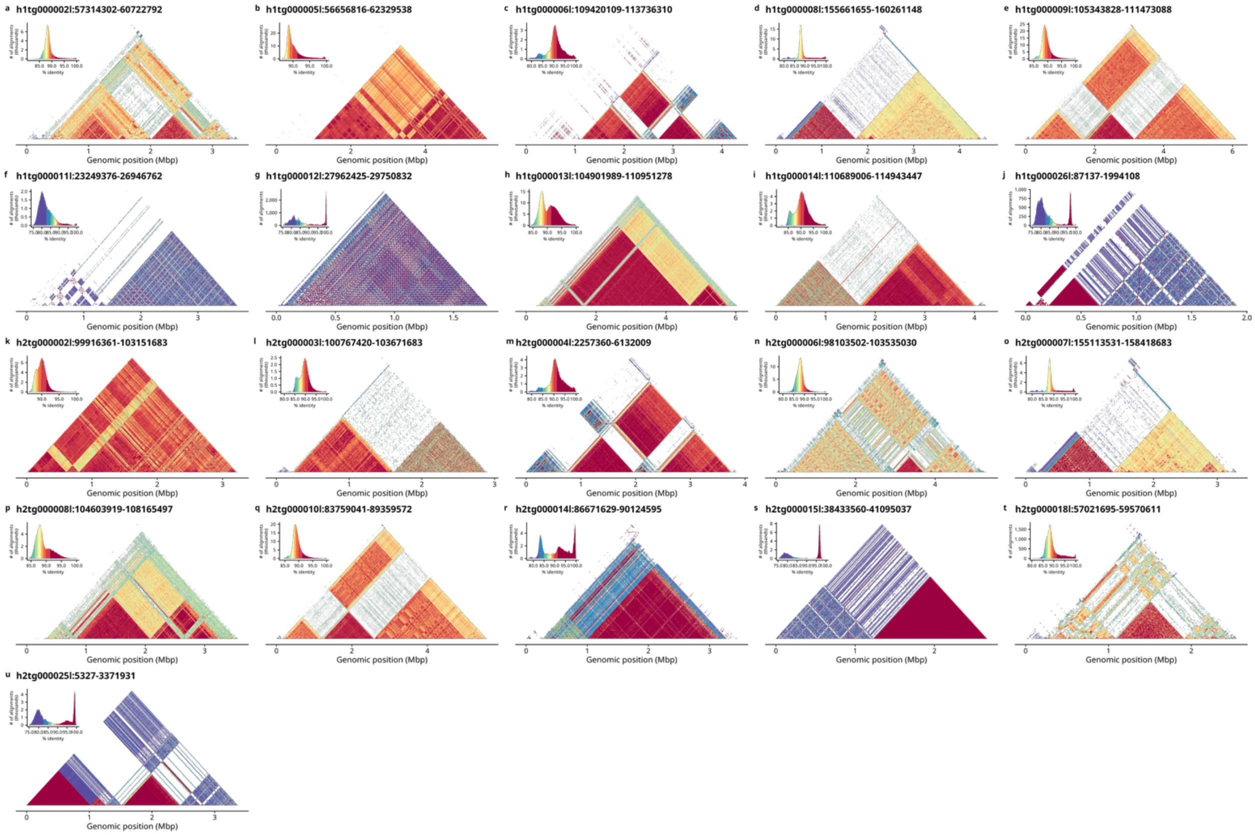

Figure S15. StainedGlass heatmaps for all the centromeres of *Varecia variegata*

bioRxiv preprint doi: https://doi.org/10.64898/2026.05.16.725662; this version posted May 19, 2026. The copyright holder for this preprint (which was not certified by peer review) is the author/funder, who has granted bioRxiv a license to display the preprint in perpetuity. It is made available under a CC-BY 4.0 International license.

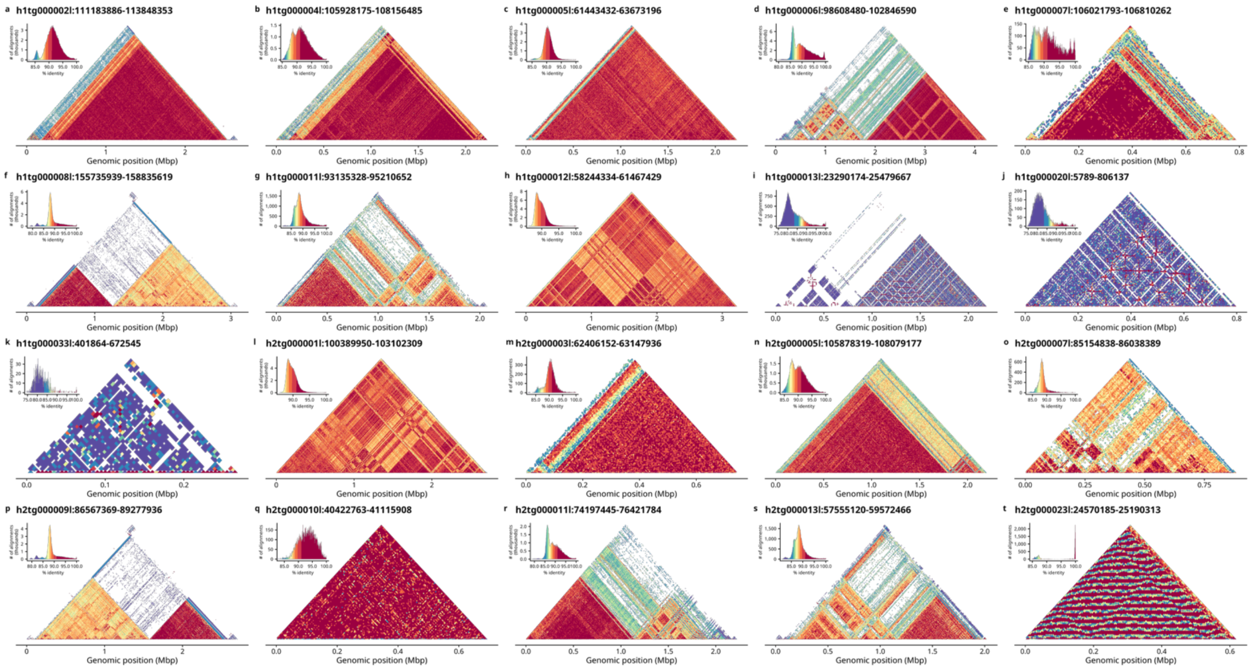

Figure S16. StainedGlass heatmaps for all the centromeres of *Varecia rubra*

bioRxiv preprint doi: https://doi.org/10.64898/2026.05.16.725662; this version posted May 19, 2026. The copyright holder for this preprint (which was not certified by peer review) is the author/funder, who has granted bioRxiv a license to display the preprint in perpetuity. It is made available under a CC-BY 4.0 International license.

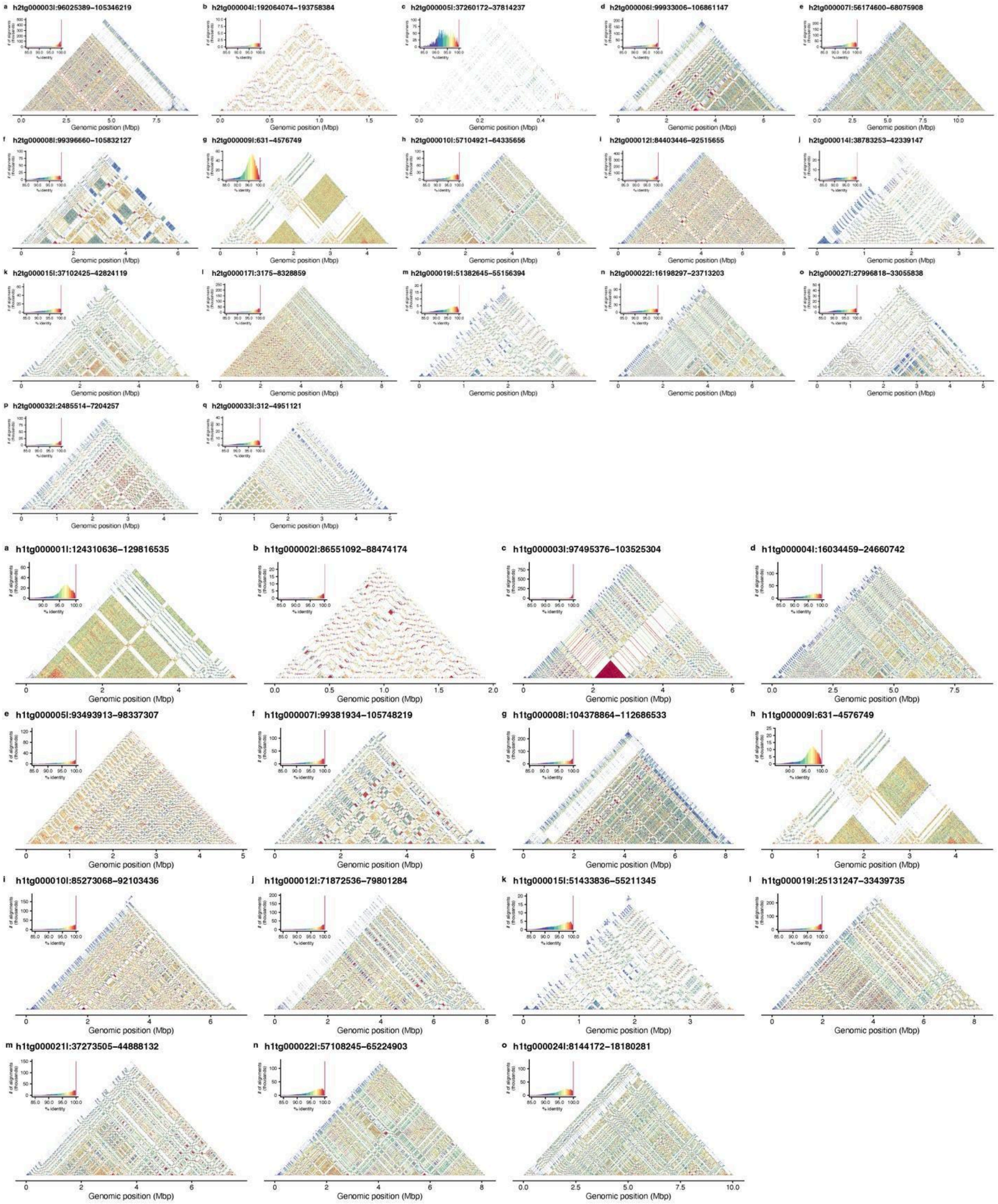

Figure S17. StainedGlass heatmaps for all the centromeres of *Eulemur collaris*

bioRxiv preprint doi: https://doi.org/10.64898/2026.05.16.725662; this version posted May 19, 2026. The copyright holder for this preprint (which was not certified by peer review) is the author/funder, who has granted bioRxiv a license to display the preprint in perpetuity. It is made available under a CC-BY 4.0 International license.

a.

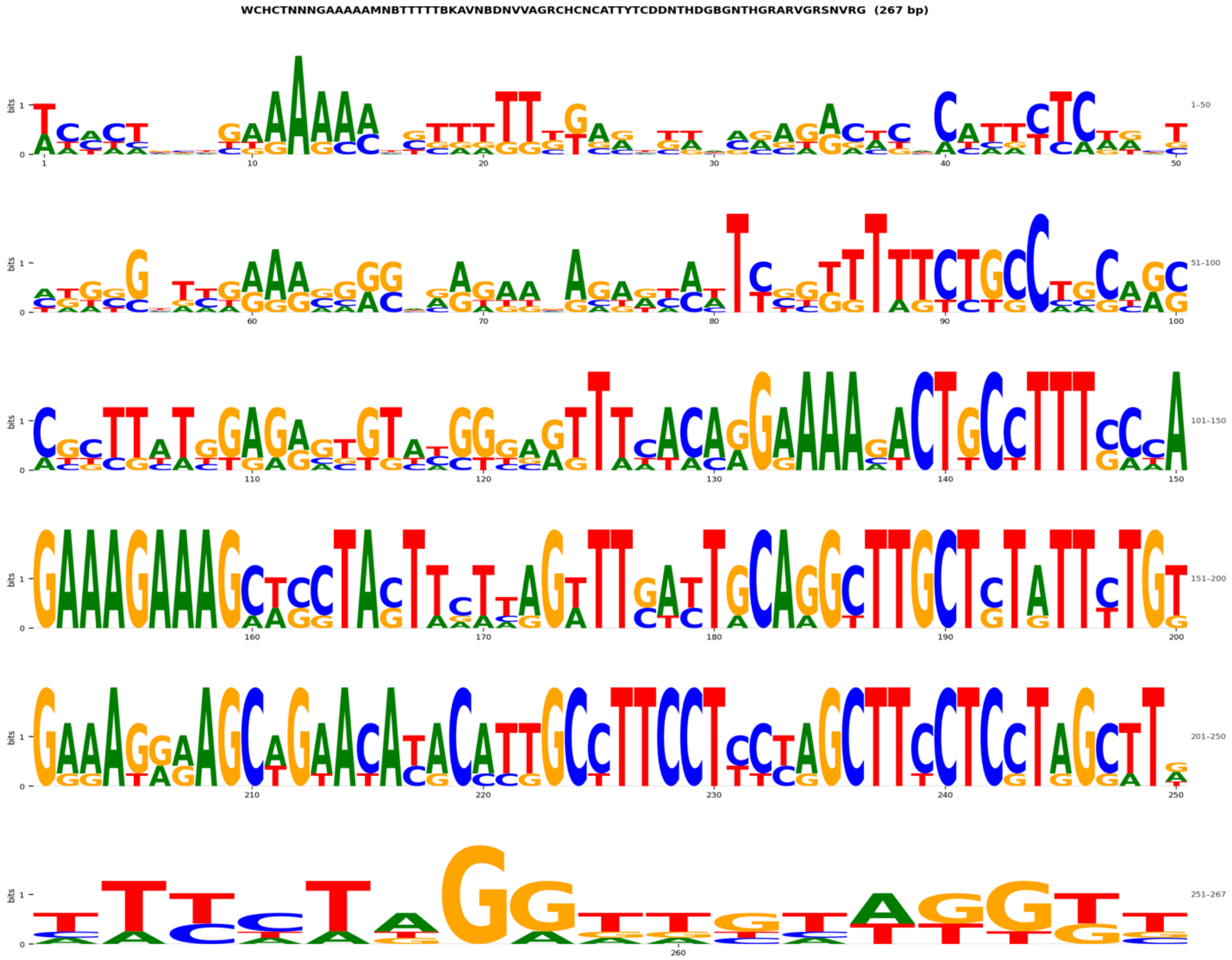

bioRxiv preprint doi: https://doi.org/10.64898/2026.05.16.725662; this version posted May 19, 2026. The copyright holder for this preprint (which was not certified by peer review) is the author/funder, who has granted bioRxiv a license to display the preprint in perpetuity. It is made available under a CC-BY 4.0 International license.

b.

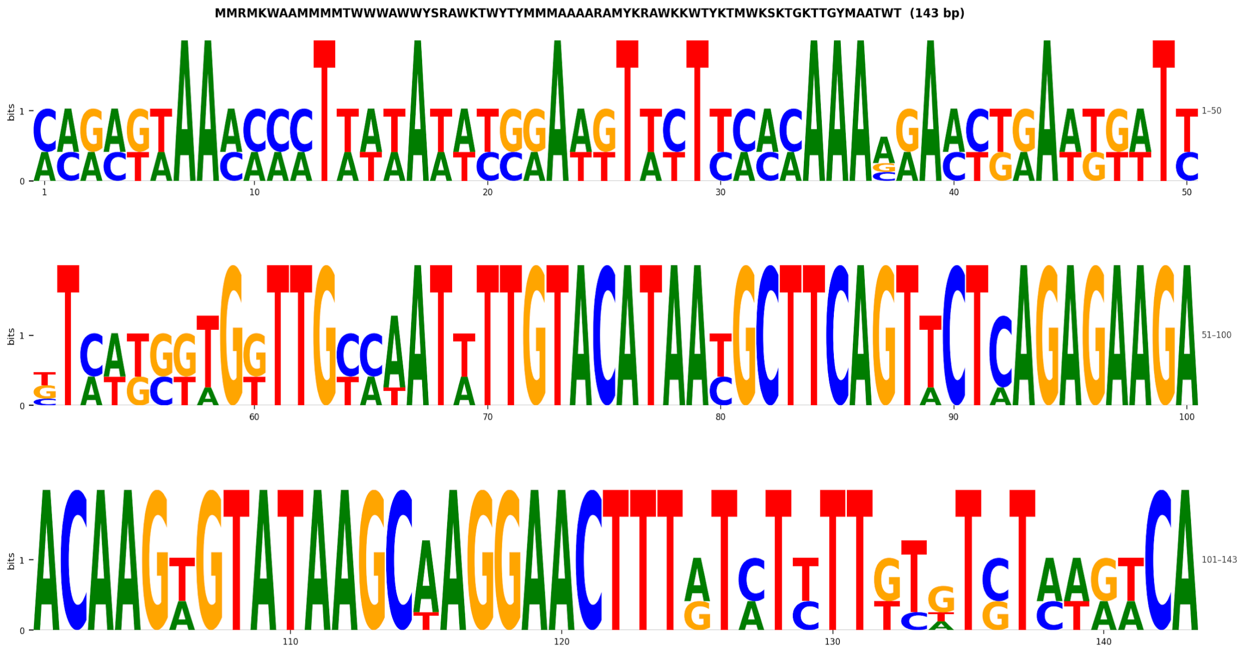

c.

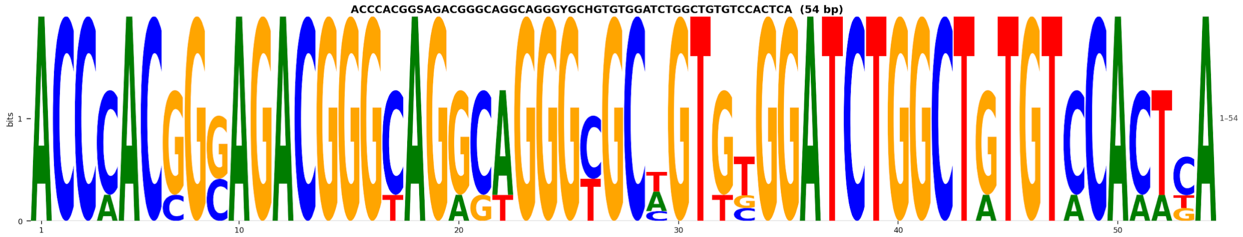

bioRxiv preprint doi: https://doi.org/10.64898/2026.05.16.725662; this version posted May 19, 2026. The copyright holder for this preprint (which was not certified by peer review) is the author/funder, who has granted bioRxiv a license to display the preprint in perpetuity. It is made available under a CC-BY 4.0 International license.

d.

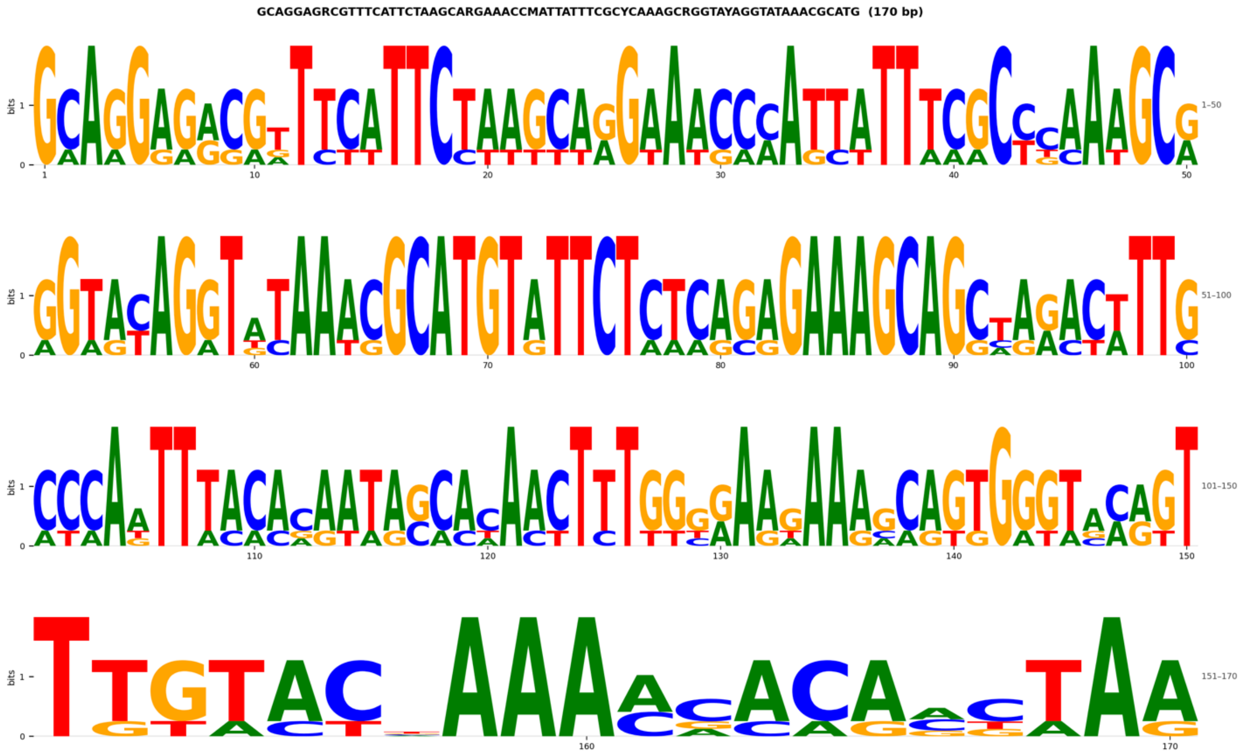

e.

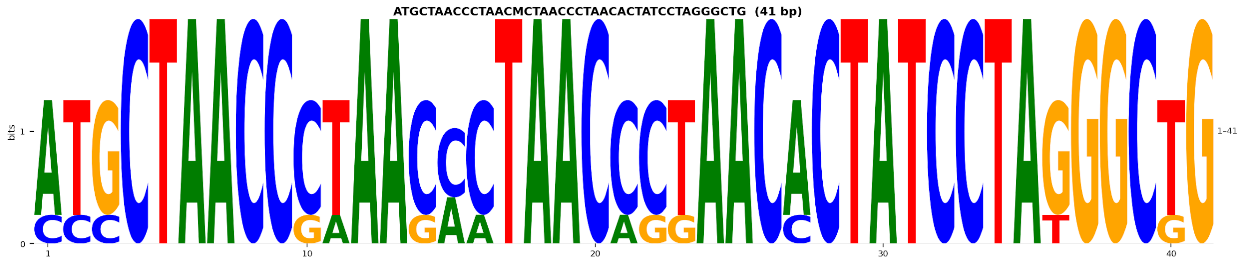

f.

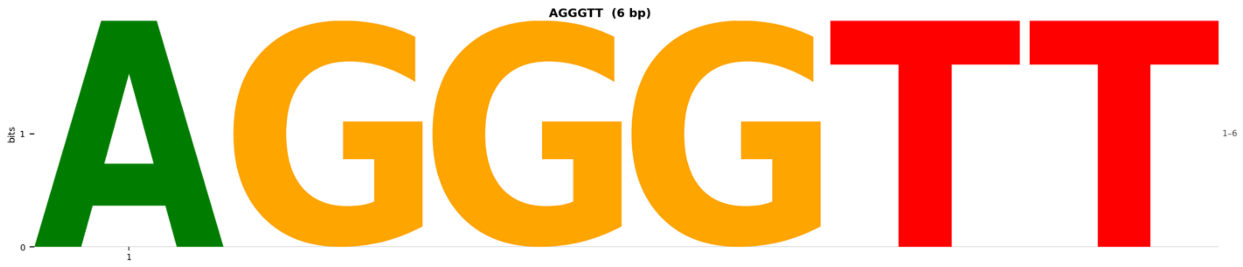

bioRxiv preprint doi: https://doi.org/10.64898/2026.05.16.725662; this version posted May 19, 2026. The copyright holder for this preprint (which was not certified by peer review) is the author/funder, who has granted bioRxiv a license to display the preprint in perpetuity. It is made available under a CC-BY 4.0 International license.

g.

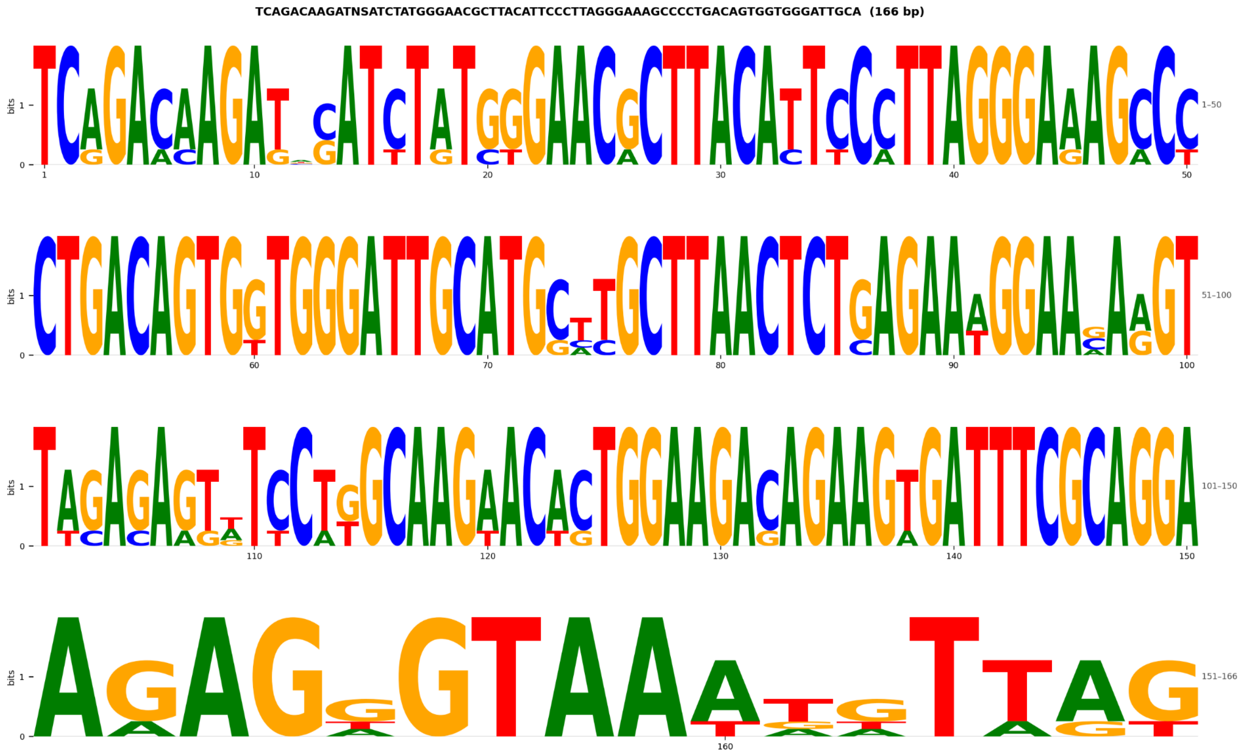

h.

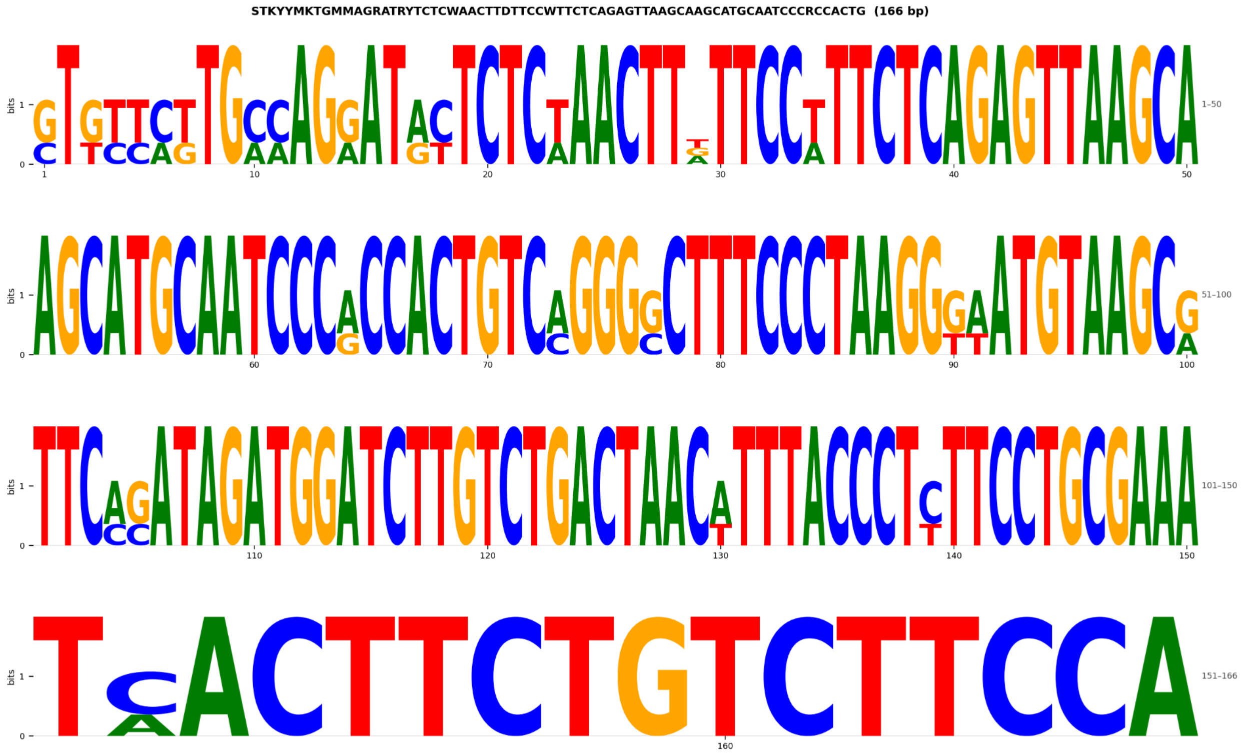

bioRxiv preprint doi: https://doi.org/10.64898/2026.05.16.725662; this version posted May 19, 2026. The copyright holder for this preprint (which was not certified by peer review) is the author/funder, who has granted bioRxiv a license to display the preprint in perpetuity. It is made available under a CC-BY 4.0 International license.

Figure S18. Logos plots for all the monomers across eight species of lemurs. Logos plots for the consensus sequences of the most common monomers in all the eight lemur species. The consensus was taken from the MEME suite, by giving a random 100 kbp centromere sequence and giving the exact monomer size. a, DMA; b, CME; c, MMU; d, PCO; e, LCA; f, ECO; g, VRU; h, VVA

bioRxiv preprint doi: https://doi.org/10.64898/2026.05.16.725662; this version posted May 19, 2026. The copyright holder for this preprint (which was not certified by peer review) is the author/funder, who has granted bioRxiv a license to display the preprint in perpetuity. It is made available under a CC-BY 4.0 International license.

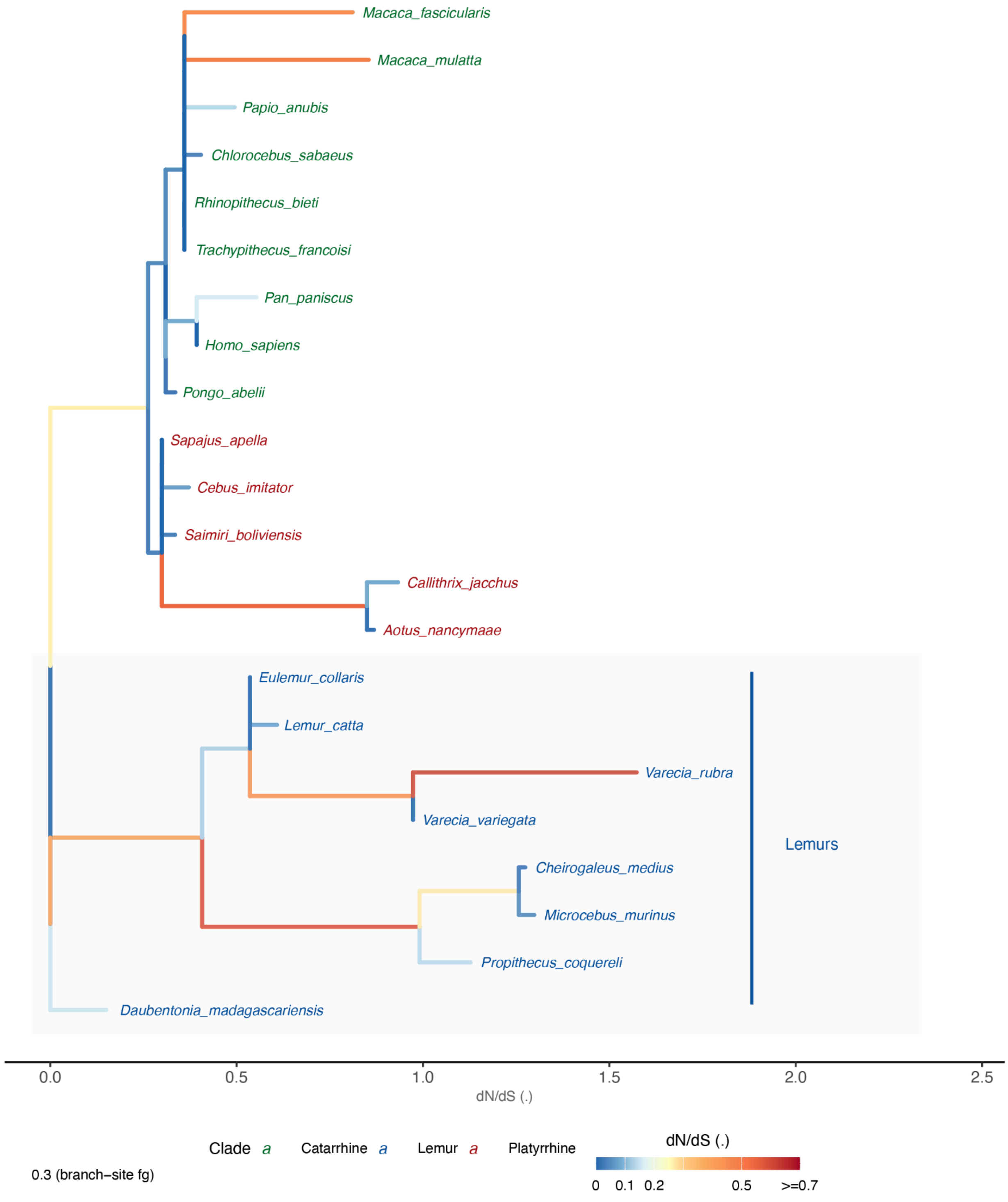

bioRxiv preprint doi: https://doi.org/10.64898/2026.05.16.725662; this version posted May 19, 2026. The copyright holder for this preprint (which was not certified by peer review) is the author/funder, who has granted bioRxiv a license to display the preprint in perpetuity. It is made available under a CC-BY 4.0 International license.

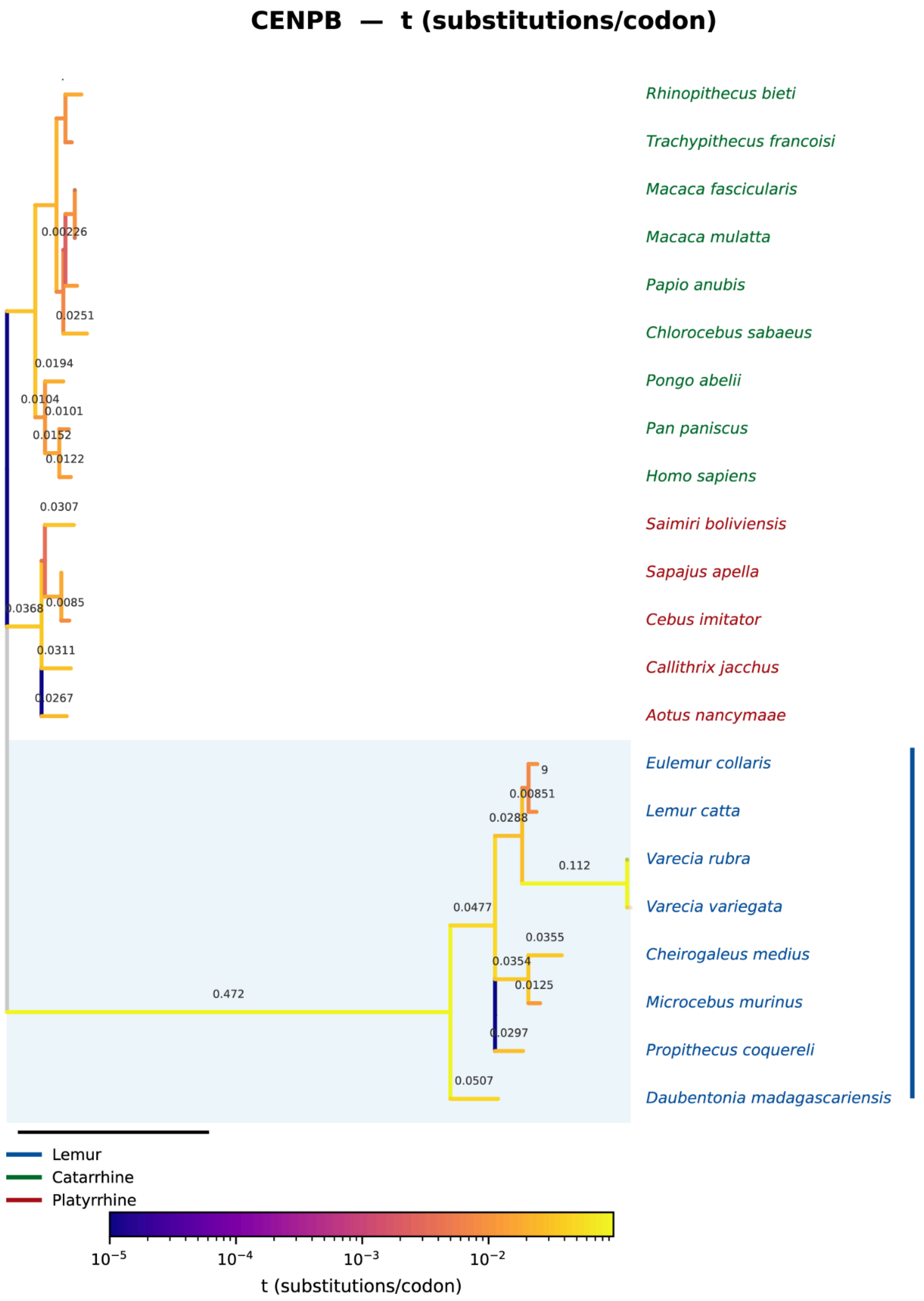

Figure S19 - Selection on CENP-B genes is depicted with two trees. a, Branch lengths are plotted with omega values showing stronger selection in lemur clade. b.

bioRxiv preprint doi: https://doi.org/10.64898/2026.05.16.725662; this version posted May 19, 2026. The copyright holder for this preprint (which was not certified by peer review) is the author/funder, who has granted bioRxiv a license to display the preprint in perpetuity. It is made available under a CC-BY 4.0 International license.

Branch length as substitutions per codon values showing the longer internal branch in lemurs.

bioRxiv preprint doi: https://doi.org/10.64898/2026.05.16.725662; this version posted May 19, 2026. The copyright holder for this preprint (which was not certified by peer review) is the author/funder, who has granted bioRxiv a license to display the preprint in perpetuity. It is made available under a CC-BY 4.0 International license.

a.

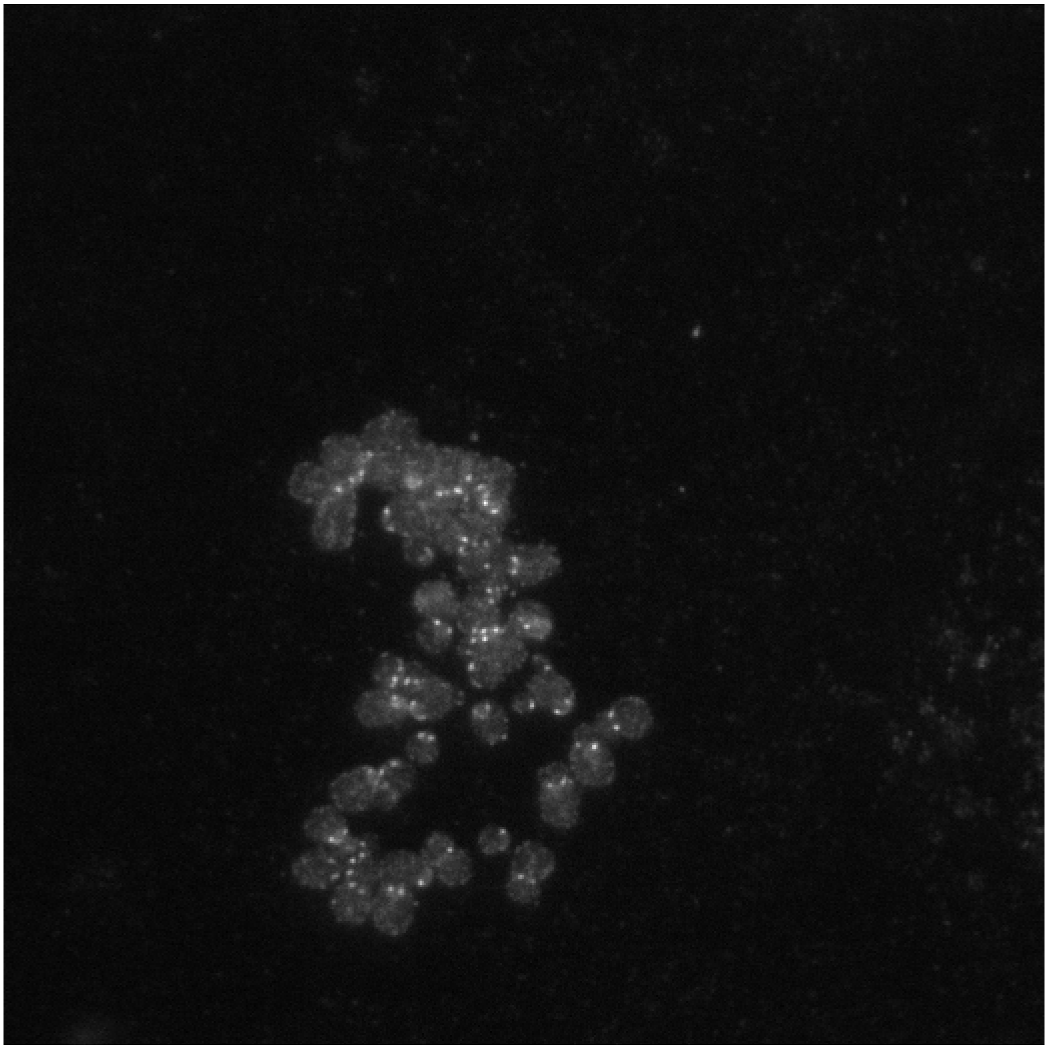

b.

bioRxiv preprint doi: https://doi.org/10.64898/2026.05.16.725662; this version posted May 19, 2026. The copyright holder for this preprint (which was not certified by peer review) is the author/funder, who has granted bioRxiv a license to display the preprint in perpetuity. It is made available under a CC-BY 4.0 International license.

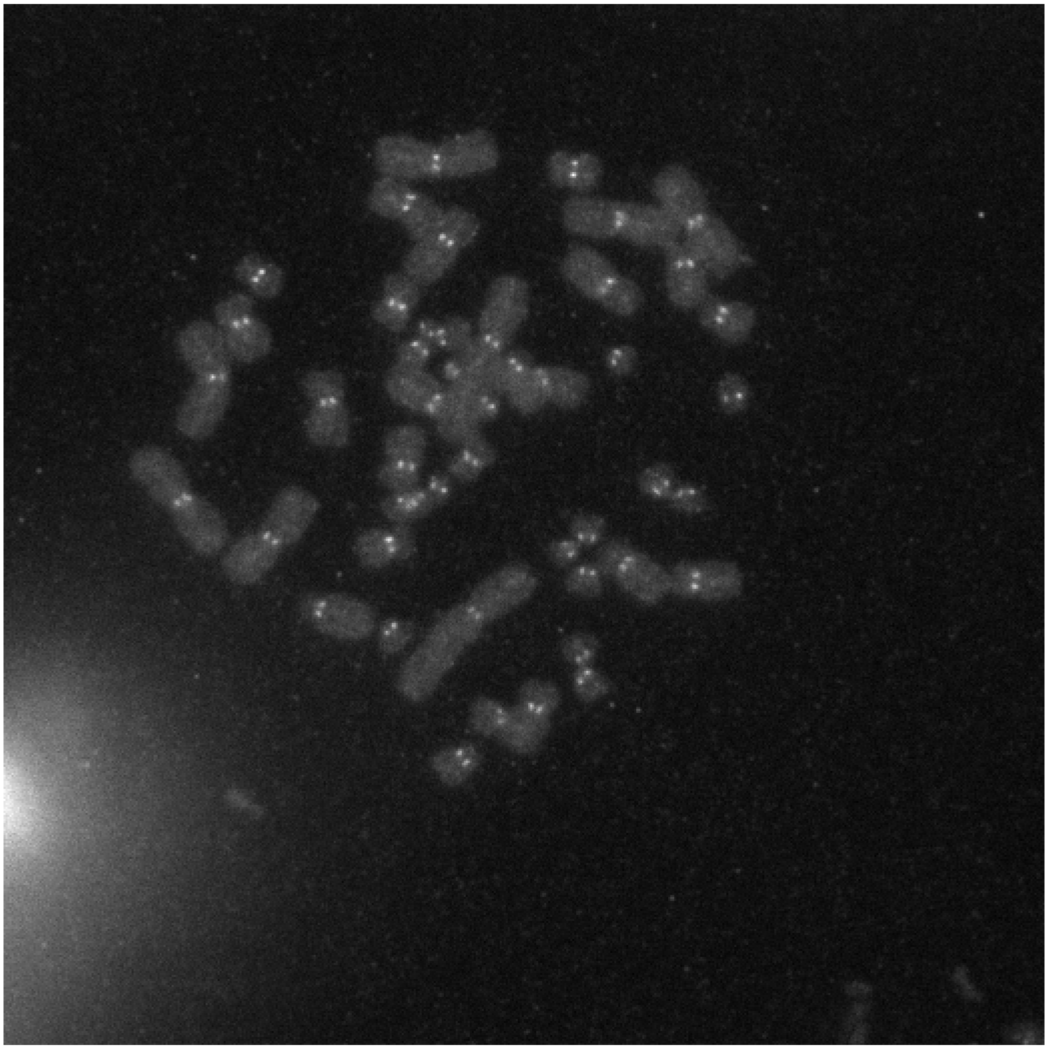

c.

bioRxiv preprint doi: https://doi.org/10.64898/2026.05.16.725662; this version posted May 19, 2026. The copyright holder for this preprint (which was not certified by peer review) is the author/funder, who has granted bioRxiv a license to display the preprint in perpetuity. It is made available under a CC-BY 4.0 International license.

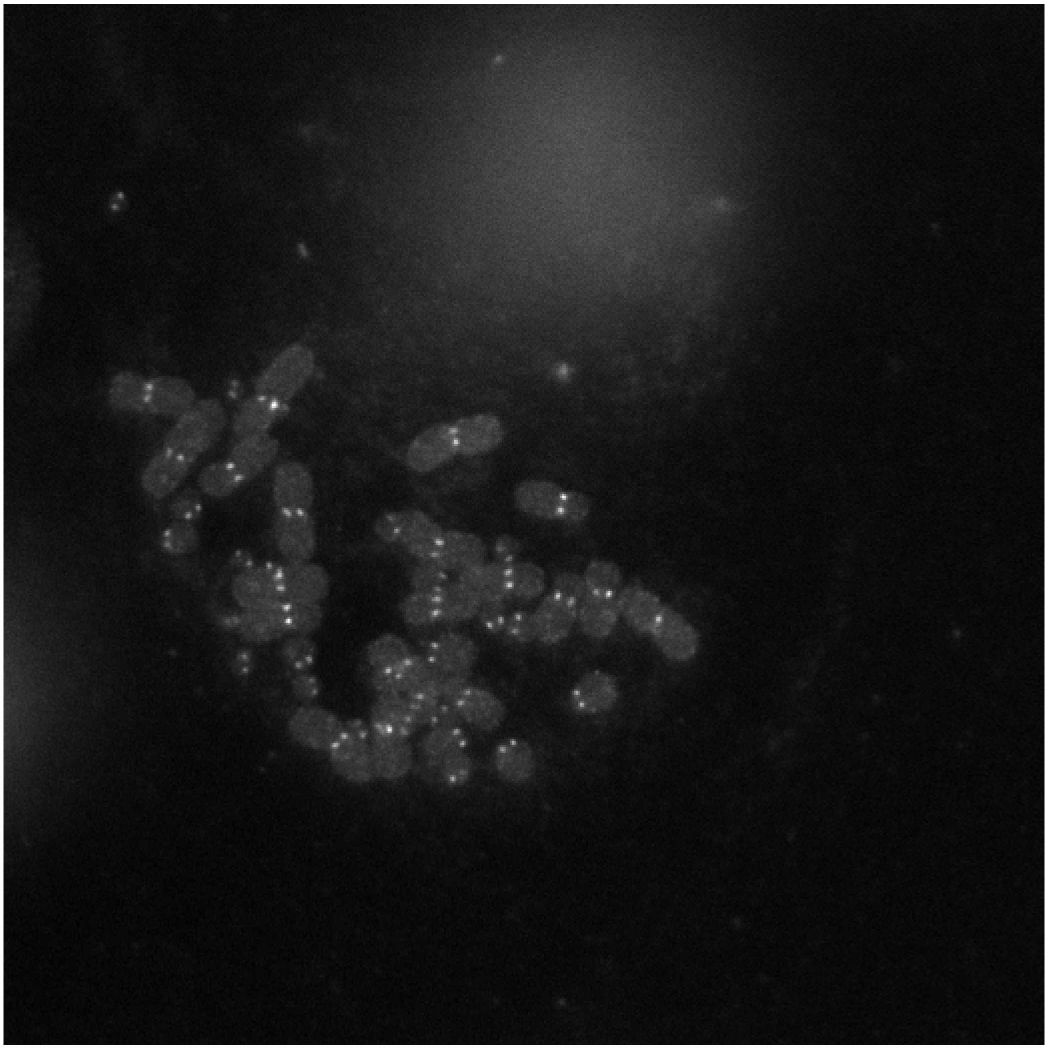

bioRxiv preprint doi: https://doi.org/10.64898/2026.05.16.725662; this version posted May 19, 2026. The copyright holder for this preprint (which was not certified by peer review) is the author/funder, who has granted bioRxiv a license to display the preprint in perpetuity. It is made available under a CC-BY 4.0 International license.

d.

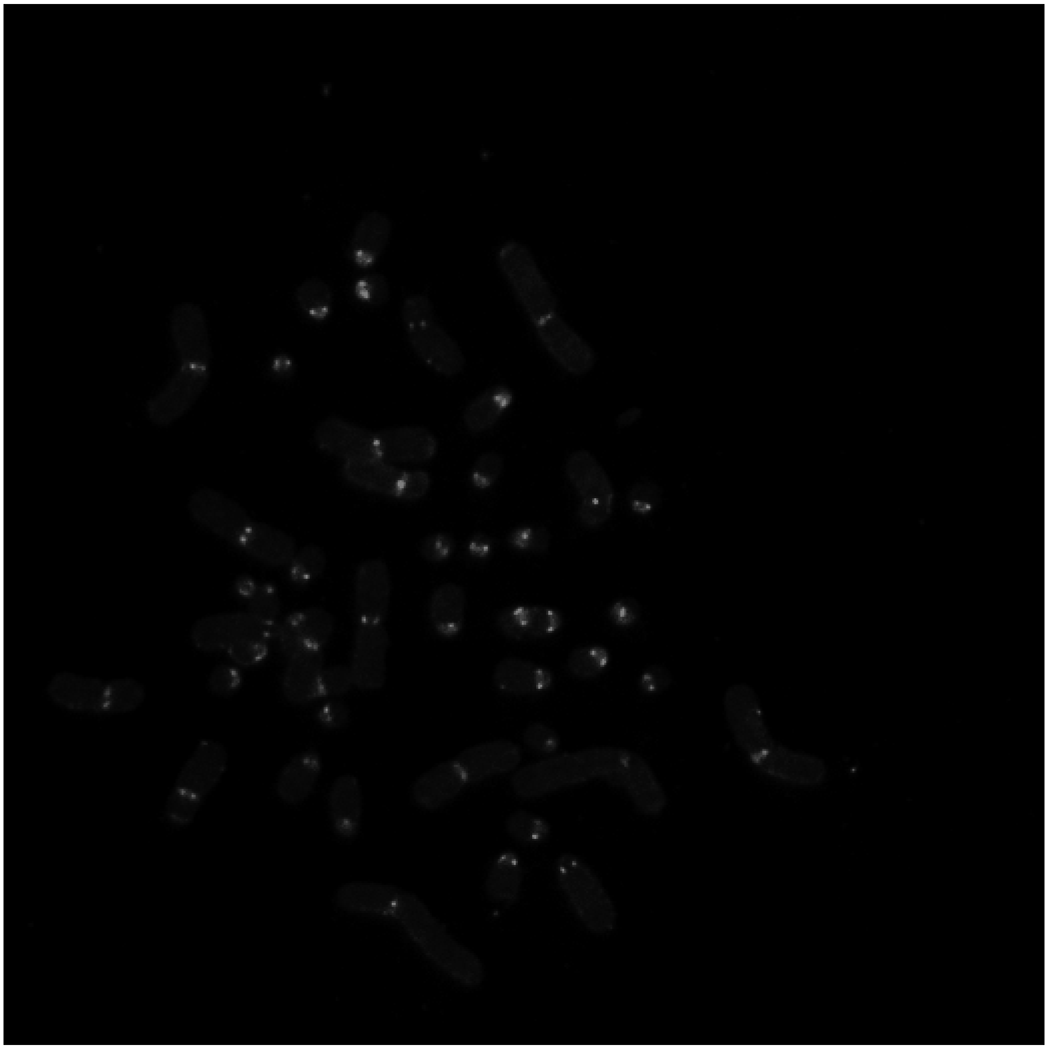

Figure S20. Raw FISH images of four species. Original raw images of FISH as seen in metaphase cells. a, LCA; b, PCO; c, VVA; d, ECI.

bioRxiv preprint doi: https://doi.org/10.64898/2026.05.16.725662; this version posted May 19, 2026. The copyright holder for this preprint (which was not certified by peer review) is the author/funder, who has granted bioRxiv a license to display the preprint in perpetuity. It is made available under a CC-BY 4.0 International license.

References

M. R. Vollger *et al.*, Long-read sequence and assembly of segmental duplications. *Nat. Methods* **16**, 88–94 (2019).

W. W. Liao *et al.*, A draft human pangenome reference. *Nature* **617**, 312–324 (2023).

M. F. Cardone, M. Ventura, S. Tempesta, M. Rocchi, N. Archidiacono, Analysis of chromosome conservation in *Lemur catta* studied by chromosome paints and BAC/PAC probes. *Chromosoma* **111**, 348–356 (2002).

N. Huang, H. Li, compleasm: a faster and more accurate reimplementation of BUSCO. *Bioinformatics* **39**, btad595 (2023).

W. Wei *et al.*, wgatools: an ultrafast toolkit for manipulating whole-genome alignments. *Bioinformatics* **41**, btaf132 (2025).

P. Wlodzimierz, M. Hong, I. R. Henderson, TRASH: Tandem Repeat Annotation and Structural Hierarchy. *Bioinformatics* **39**, btad308 (2023).

T. L. Bailey, J. Johnson, C. E. Grant, W. S. Noble, The MEME Suite. *Nucleic Acids Res.* **43**, W39–W49 (2015).

F. Kumara Mastrorosa *et al.*, Identification and annotation of centromeric hypomethylated regions with CDR-Finder. *Bioinformatics* **40**, btae733 (2024).

S. Gao *et al.*, HiCAT: a tool for automatic annotation of centromere structure. *Genome Biol.* **24**, 58 (2023).

G. A. Logsdon *et al.*, The variation and evolution of complete human centromeres. *Nature* **629**, 136–145 (2024).

M. R. Vollger, P. Kerpedjiev, A. M. Phillippy, E. E. Eichler, StainedGlass: interactive visualization of massive tandem repeat structures with identity heatmaps. *Bioinformatics* **38**, 2049–2051 (2022).

Z. Yang, PAML 4: phylogenetic analysis by maximum likelihood. *Mol. Biol. Evol.* **24**, 1586–1591 (2007).

W. Shen, B. Sipos, L. Zhao, SeqKit2: a Swiss army knife for sequence and alignment processing. *iMeta* **3**, e191 (2024).

J. Dainat, AGAT: Another GFF Analysis Toolkit to handle annotations in any GTF/GFF format (Version v0.8.0). Zenodo. https://doi.org/10.5281/zenodo.3552717 (2021).

K. Katoh, J. Rozewicki, K. D. Yamada, MAFFT online service: multiple sequence alignment, interactive sequence choice and visualization. *Brief. Bioinform.* **20**, 1160–1166 (2019).

M. Suyama, D. Torrents, P. Bork, PAL2NAL: robust conversion of protein sequence alignments into the corresponding codon alignments. *Nucleic Acids Res.* **34**, W609–W612 (2006).

S. Kumar *et al.*, TimeTree 5: an expanded resource for species divergence times. *Mol. Biol. Evol.* **39**, msac174 (2022).

bioRxiv preprint doi: https://doi.org/10.64898/2026.05.16.725662; this version posted May 19, 2026. The copyright holder for this preprint (which was not certified by peer review) is the author/funder, who has granted bioRxiv a license to display the preprint in perpetuity. It is made available under a CC-BY 4.0 International license.

J. O. Wertheim *et al.*, RELAX: detecting relaxed selection in a phylogenetic framework. *Mol. Biol. Evol.* **32**, 820–832 (2015).

B. Murrell *et al.*, Gene-wide identification of episodic selection. *Mol. Biol. Evol.* **32**, 1365–1371 (2015).
